# Ductal Carcinoma in Situ: Molecular Changes Accompanying Disease Progression

**DOI:** 10.1007/s10911-022-09517-7

**Published:** 2022-05-14

**Authors:** Gemma M. Wilson, Phuong Dinh, Nirmala Pathmanathan, J. Dinny Graham

**Affiliations:** 1 Centre for Cancer Research, The Westmead Institute for Medical Research, The University of Sydney, Westmead, NSW 2145 Australia; 2 Westmead Breast Cancer Institute, Westmead Hospital, Westmead, NSW 2145 Australia

**Keywords:** Ductal carcinoma in situ, Microenvironment, Cancer progression, Immune infiltration

## Abstract

Ductal carcinoma in situ (DCIS) is a non-obligate precursor of invasive ductal carcinoma (IDC), whereby if left untreated, approximately 12% of patients develop invasive disease. The current standard of care is surgical removal of the lesion, to prevent potential progression, and radiotherapy to reduce risk of recurrence. There is substantial overtreatment of DCIS patients, considering not all DCIS lesions progress to invasive disease. Hence, there is a critical imperative to better predict which DCIS lesions are destined for poor outcome and which are not, allowing for tailored treatment. Active surveillance is currently being trialed as an alternative management practice, but this approach relies on accurately identifying cases that are at low risk of progression to invasive disease. Two DCIS-specific genomic profiling assays that attempt to distinguish low and high-risk patients have emerged, but imperfections in risk stratification coupled with a high price tag warrant the continued search for more robust and accessible prognostic biomarkers. This search has largely turned researchers toward the tumor microenvironment. Recent evidence suggests that a spectrum of cell types within the DCIS microenvironment are genetically and phenotypically altered compared to normal tissue and play critical roles in disease progression. Uncovering the molecular mechanisms contributing to DCIS progression has provided optimism for the search for well-validated prognostic biomarkers that can accurately predict the risk for a patient developing IDC. The discovery of such markers would modernize DCIS management and allow tailored treatment plans. This review will summarize the current literature regarding DCIS diagnosis, treatment, and pathology.

## Introduction

**Fig. 1:**
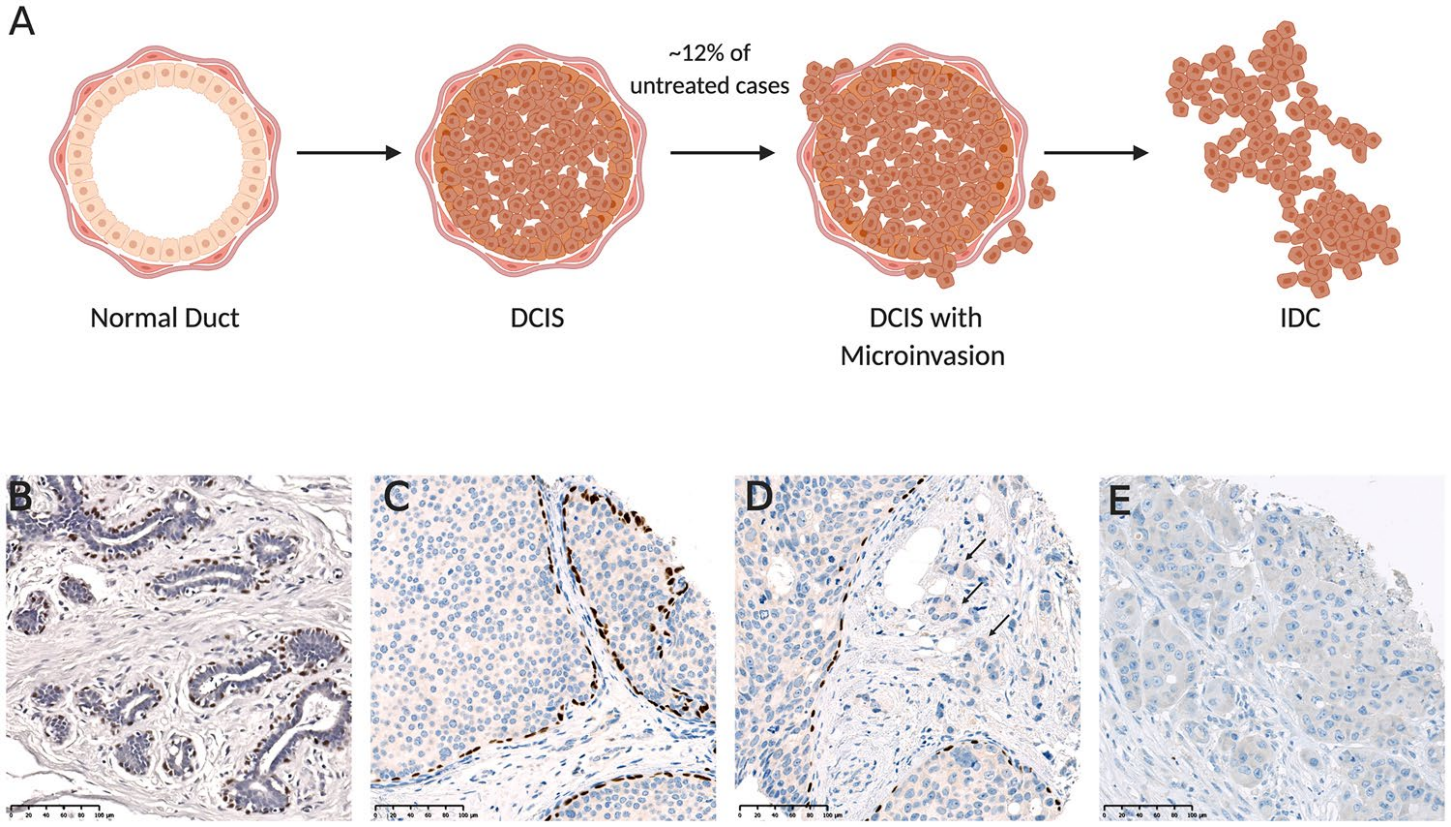
Ductal carcinoma in situ progression. **A** Progression of normal tissue to ductal carcinoma in situ, to ductal carcinoma in situ with microinvasion, to invasive ductal carcinoma. Drawing created with BioRender.com. **B**-**E** Immunoperoxidase staining of myoepithelial marker, p63 in: **B** Normal breast tissue, **C** ductal carcinoma in situ, **D** ductal carcinoma in situ with microinvasion (indicated by arrows), and **E** invasive ductal carcinoma. Scale bar representative of 100 µm.

Ductal Carcinoma in Situ (DCIS) refers to the non-invasive proliferation of neoplastic epithelial cells confined within the ducts of the breast. In the normal breast, the mammary gland consists of a ductal-lobular branching system lined by an inner luminal epithelial cell layer and an outer layer of contractile myoepithelial cells [1]. Given that the neoplastic epithelial cells are confined within the lumen of the duct by a layer of myoepithelial cells surrounded by a basement membrane, DCIS lesions are considered non-invasive [2] (Fig. 1). Due to the increasing accessibility of mammographic screening programs, the incidence of DCIS is rising, with an estimated 48,530 new DCIS cases in the USA in 2020 [3]. Although non-invasive, DCIS is a non-obligate precursor of invasive ductal carcinoma (IDC) whereby the neoplastic epithelial cells may evade ductal confinement, breach the myoepithelium and basement membrane, and invade surrounding tissue (Fig. 1).

Compared to the age-adjusted average 10-year risk of a new diagnosis of DCIS of less than 3% [3, 4], a woman’s risk of a subsequent DCIS or invasive breast cancer diagnosis is considerably increased regardless of clinical management. According to a recent review of women in the US National Cancer Institute’s Surveillance, Epidemiology, and End Results (SEER) program (1992–2014), who were diagnosed with DCIS and did not receive any locoregional treatment, the 10-year overall risk of ipsilateral IDC was 12.1% [5]. Currently, the progression and recurrence of DCIS is clinically unpredictable. The current standard of care for all patients involves surgical removal of the lesion, generally followed by radiation treatment of the affected breast, and in some cases adjuvant endocrine therapy [6]. In a study by the Early Breast Cancer Triallists Collaborative Group, which combined the results of four randomized trials (NSABP B-17, EORTC 10,853, SweDCIS, UK/ANZ DCIS), spanning 1985 to 2000, comparing surgery alone to surgery plus adjuvant radiation, the risk of any ipsilateral recurrence (either invasive or in situ) if treated with surgery alone was 28.1% [7]. Half (48.3%) of these recurrences were IDC, placing the risk of invasive recurrence at 13.6%, close to that observed with no surgery in the SEER study. Although this rate is potentially higher than would be expected in a more recent cohort, it is not dissimilar to other estimates of recurrence after surgery alone [8]. Adjuvant radiation therapy halved the risk of any ipsilateral recurrence, but did not have a significant impact on breast cancer-specific mortality [7].

Since up to 88% of untreated cases will remain in situ and potentially not progress, there is considerable overtreatment of DCIS patients. Standard locoregional breast cancer treatment (lumpectomy and radiation) costs the US Medicare system an estimated $10,538 per patient [9], equating to a cost of over $500 million for the primary management of all DCIS cases diagnosed in the US in 2020. The overtreatment of screen-detected yet often asymptomatic DCIS mandates research into better and more affordable approaches to more accurately determine whether DCIS is likely to progress or remain in a pre-invasive state. Accurate risk stratification would enable personalized treatment of DCIS patients, rather than a ‘one size fits all’ approach, which would alleviate the physical, mental, and financial burden on patients and health care systems. However, the ability to accurately predict the risk of DCIS progression requires a profound understanding of the complex interactions that contribute to DCIS progression and recurrence.

## Diagnosing DCIS

The advent of nationwide mammographic screening programs has resulted in a substantial worldwide increase in breast cancer diagnoses [10]. Today, DCIS accounts for over 20% of screen detected breast lesions, whereas prior to screening programs DCIS made up just 3% of breast cancer diagnoses [2, 11]. Up to 98% of DCIS lesions are asymptomatic and non-palpable, with clinical symptoms only reported in a minority of cases [2]. In fact, the extent of tumor cells is often too small to be identified directly on mammography and consequently, up to 90% of DCIS diagnoses are due to calcifications identified by mammography [2]. Although calcifications are common and frequently benign, certain patterns of calcifications such as fine linear or branching morphology can be indicative of malignancies such as DCIS [12]. Core biopsies are frequently used to establish a diagnosis with indeterminate calcifications. A recent study of DCIS cases recorded in the US National Cancer Institute’s SEER registries database between 1995 and 2014 revealed a median lesion size of 1.1 cm [13]. However, tumors can be considerably more extensive on diagnosis, with 4.8% of cases presenting with regions of DCIS greater than 5 cm [13]. Depending on the degree of nuclear atypia, polarization, presence of necrosis and architectural pattern, DCIS is classified as low (well differentiated), intermediate (moderately differentiated) or high (poorly differentiated) grade [14, 15]. Histologically, DCIS is a malignant epithelial proliferation that is confined to a duct. Immunohistochemical makers for myoepithelial cells such as CD10, smooth muscle actin (SMA) and p63 (Table 1), can be used to confirm the presence of the myoepithelial cell layer around involved ducts. A diagnosis of pure DCIS is only made in cases where the myoepithelial layer is intact, with no evidence of invasion into the surrounding basement membrane. In cases where DCIS is accompanied by small islands of tumor cells adjacent to the DCIS foci, a diagnosis of DCIS with microinvasion is made. These cases, which make up around 3% of DCIS diagnoses, are considered to be quite different from pure DCIS and are managed similarly to early stage invasive disease [16]. In cases where both DCIS and invasive disease are present in the one lesion, the primary diagnosis is invasive breast cancer which drives the prognosis and treatment pathway.

**Table 1:** Staining pattern and distribution of common myoepithelial cell markers in the breast. Adapted from [95, 123–125]

**Marker**	**Staining pattern**	**Expression score**			
		**Myoepithelial cell**	**Fibroblast**	**VSMC**	**Tumor cell**
α-SMA	Cytoplasmic	3 +	3 +	3 +	0
Calponin	Cytoplasmic	3 +	1 +	2 +	1 +
CD10	Membranous	2 +	1 +	0	1 +
P63	Nuclear	3 +	0	0	1 +
SMMHC	Cytoplasmic	3 +	1 +	2 +	0

*VSMC* Vascular smooth muscle cell. Expression score (frequency and intensity): 0 = Not Detected, 1 +  = Low, 2 +  = Intermediate, 3 +  = High

## The Natural Course of DCIS

Given that most DCIS is surgically resected, the natural course of the disease, including factors influencing its progression, are difficult to evaluate [2]. However, a handful of retrospective studies have reported the natural course of DCIS in patients where there was no locoregional treatment after initial diagnosis, either due to misdiagnosis as a benign lesion [17–20] or in older patients where other factors prevented surgery [5]. The smaller studies that reviewed disease course after misdiagnosis reported that anywhere between 14 to 53% of patients went on to develop IDC within 10 years [17–20]. While patients with high grade DCIS were more likely to develop IDC, non-high grade DCIS also progressed to invasive disease during the period. However, these proportions are difficult to relate to the normal population of patients with DCIS, given that they are derived from small cohorts of misdiagnosed cases with unknown extent of disease at the time of biopsy. In a larger review of 1286 patients who did not receive surgery or radiation treatment for a primary diagnosis of DCIS, as recorded in the SEER program (1992–2014), the 10-year net risk of ipsilateral IDC was 12.2% in patients with non-high grade DCIS and 17.6% in patients with high grade disease. The 10 year cumulative incidence of ipsilateral invasive breast cancer over the whole cohort was 10.5% and the incidence of contralateral invasive disease was 3.9% [5]. These data suggest that, while the risk of progression to invasive disease is not insignificant, there is considerable over-treatment of DCIS, particularly in older patients. The 10-year cumulative all-cause mortality rate among untreated patients was substantially higher than that of DCIS patients who were treated according to standard guidelines (24.1% compared to 12.1%), although it is unlikely this disparity was a result of the untreated DCIS alone. High mortality rates in the untreated group were possibly due to a higher level of additional comorbidities, as well as differential screening and surveillance practices. Consequently, the impact of omitting treatment for DCIS, on disease-specific mortality, could not be accurately estimated in this cohort.

## The Overtreatment of DCIS

### Surgery

The prognostic uncertainty of DCIS means 98% of patients receive locoregional treatment involving surgery, with or without radiation treatment [6]. Surgical procedures to remove the tumor vary from breast conserving surgery (BCS) to mastectomy, depending on the extent of the DCIS, predicted risk of recurrence and patient preference. Although surgical intervention mitigates the risk of invasive progression of the primary tumor, a risk remains for recurrence. One study examined the risk of ipsilateral breast events for non-high grade and high grade DCIS in patients selected to undergo BCS alone based on perceived low risk clinical and pathological characteristics [8]. While the 5-year recurrence rate for low-intermediate grade DCIS tumors, with a diameter of 2.5 cm or smaller, was just 6% and the recurrence rate for small high grade DCIS tumors (diameter of 1 cm or less) was 15%, the recurrence rate increased steadily in both groups over the 12 year period without plateau, such that after 12 years, the recurrence rate was 14.4% for the non-high grade cohort and 24.6% for the high grade cohort. Moreover, 50% of ipsilateral tumors recurred as invasive cancer regardless of the grade of the original DCIS [8]. These findings suggest that, even in DCIS assessed as very low risk based on clinical and pathological features, the risk of ipsilateral recurrence and development of invasive disease are unpredictable and continue to rise over time, with no evidence of levelling out over longer periods. This is an important consideration for physicians, particularly when treating younger patients, and leads to almost universal adjuvant management.

### Radiation

Four early randomized trials revealed that recurrence rates are significantly reduced by the addition of radiotherapy after conservative surgery for DCIS [21–24]. The prospective studies occurred over a period of up to 20 years, with the long-term risks of recurrent breast disease after surgery alone or surgery plus radiation published between 2011–2014 [21–24]. In all studies, long term follow-up supported the use of radiotherapy after BCS, with an approximate halving of the risk of an ipsilateral breast cancer recurrence in patients receiving radiotherapy, compared with patients treated with BCS alone [21–24]. Since then, many other prospective studies have assessed the benefit of radiotherapy after surgery and have confirmed these findings [8, 21–29], emphasizing the importance of radiotherapy after surgery for DCIS patients. However, given relatively low recurrence rates, there is still substantial overuse of radiotherapy, which results in short- and long-term toxicities and a cost to the patient’s broader health. For instance, the proportion of patients suffering mild (grade 1) and moderate (grade 2) acute toxicities [30] increased by 46% in one study [26]. While the main toxic effects were not disclosed, mild and moderate toxicities from radiotherapy include skin reactions, swelling, pain, tiredness and fatigue, and lymphoedema [30]. Moreover, while recurrence rates are reduced by radiotherapy, there is no improvement in breast cancer-specific mortality associated with radiotherapy treatment [27]. In fact, in the National Surgical Adjuvant Breast and Bowel Project (NSABP) study, 15-year breast cancer-specific mortality was slightly higher in patients who received radiotherapy (4.7%) than patients who received surgery only (3.1%), although this difference was not significant [23]. Despite these consequences, higher recurrence rates after BCS alone, relative to BCS + radiotherapy, combined with a 50% risk that the recurrence will be invasive, drives a reluctance to advise against radiotherapy. Improved risk stratification is needed to more accurately predict which patients are at elevated risk of recurrence and thus would benefit from radiotherapy, and those who could be spared.

### Hormone Therapy

For the 70–80% of ER + invasive early breast cancers, treatments targeting the estrogen signaling pathway (either the antiestrogen tamoxifen which binds and inhibits ER, or aromatase inhibitors that block estrogen production) are the standard of care in the adjuvant setting for the majority of patients [31]. Moreover, treating women who are at high risk of developing breast cancer with prophylactic tamoxifen significantly reduces the risk of developing ER + disease. While around 70% of DCIS lesions are ER + [32], adjuvant tamoxifen therapy is not standard treatment. This is partly due to the assumed low risk of recurrence, but also because there is not enough evidence to prove it’s benefit. Two of the early prospective studies tested the benefit of endocrine therapy with tamoxifen on reducing the risk of local recurrence for DCIS patients [21, 23]. The NSABP B-24 double blinded trial compared recurrence rates in DCIS patients who received lumpectomy followed by radiotherapy (LRT) plus placebo or LRT plus 5 years of tamoxifen [23]. There was a 32% decrease in the risk of an invasive local recurrence in patients receiving LRT + tamoxifen compared to LRT + placebo that was statistically significant and additive with the reduction in risk from radiation therapy. However, the reduced risk equated to a very small decrease in actual recurrences at 15 years that could be attributed to benefits from tamoxifen (8.5% for LRT + tamoxifen compared to 10% in the placebo control) [23]. There was a 19% reduction in breast cancer specific mortality risk with tamoxifen compared to placebo [23]. However, this difference was not significant, due to the very low 15-year mortality rates of 2.7% for LRT + placebo treated compared with 2.3% for LRT + tamoxifen treated patients, respectively. In terms of contralateral breast events, they found a 32% reduction in risk for patients receiving LRT + tamoxifen compared to those receiving LRT + placebo, in agreement with the known benefit in reducing the risk of new primary lesions [23]. Cuzick and colleagues found that tamoxifen treatment alone after BCS reduced the 10-year risk of all new breast events by 6.5% [21]. However, for patients who also received RT, there was no added benefit of tamoxifen in reducing the risk of any new breast event [21]. In summary, while tamoxifen therapy reduced the recurrence rate for DCIS after surgery for DCIS, the benefit is small, and these studies favor local over systemic treatment in reducing the risk of recurrence. Given the already low risk of recurrence in DCIS when managed with surgery and radiotherapy, the side effects of endocrine treatment as well as the difficulty of ensuring patient compliance in taking the oral medication diminish its use in this setting.

### Active Surveillance as an Alternative to Surgery and RT

There are reservations regarding the de-escalation of the treatment of DCIS. These reservations are supported by evidence showing there is little to distinguish DCIS from invasive disease at the genomic level [33], suggesting that all DCIS has the potential to progress given sufficient time. To investigate potential strategies to de-escalate treatment of low-risk DCIS, several prospective studies are currently comparing standard DCIS treatment with active surveillance [34–38]. An outline of the LORIS, LORD, COMET, LORETTA, and LARRIKIN trials, including their inclusion and exclusion criteria, are presented in Table 2. The results of these prospective studies will reveal the feasibility of replacing upfront surgery with active surveillance, in the form of regular mammograms, for some DCIS patients. If successful, this approach may be incorporated into the clinical setting and have the potential to reduce surgery rates for new DCIS patients who are considered at low risk of progression to invasive breast cancer. However, a pre-operative in situ diagnosis by biopsy is sometimes upstaged to an invasive diagnosis upon post-operative pathological review. Since active surveillance precludes surgical excision, there is a possibility that small invasive lesions may go undiagnosed and may increase mortality rates in the long term. These trials require vacuum assisted core biopsies as opposed to narrower bore core needle biopsies to reduce this possibility, however the upstaging rate of DCIS lesions diagnosed by this approach is as high as 27.1% [39]. Moreover, one study reported that 6%, 7% and 10% of COMET, LORIS and LORD eligible patients, respectively, were upstaged to a classification of invasive disease after surgery [40]. One study examining LORIS-eligible DCIS patients undergoing surgery found that one fifth of patients were post-operatively upstaged to invasive carcinoma, and of these cases, 20% were high grade or featured lymph node involvement [41]. Another study found that 19% of all upstaged DCIS cases met LORIS criteria, however, just 3% of upstaged cases met the LORD criteria, which can be largely attributed to the fact that the LORD criteria limits to low grade DCIS while the LORIS scheme includes both low and intermediate grade DCIS [42]. In summary, active surveillance is an ideal approach for managing some DCIS patients, but its efficacy is reliant on accurate diagnosis by biopsy and identification of low-risk DCIS patients. Furthermore, this approach requires a high standard of regular mammographic surveillance, which requires high levels of patient compliance and diagnostic expertise. Nevertheless, the long-term results of these trials will prove beneficial in further delineating the natural progression of DCIS and may be helpful in identifying more robust prognostic biomarkers.

**Table 2:** Summary of current active surveillance trials

	LORIS (UK) [34]	LORD (Netherlands) [35]	COMET (USA) [36]	LORETTA (Japan) [37]	LARRIKIN (AUS & NZ) [38]
Recruitment start date	2014	2017	2017	2017	n/s
Follow-up (years)	10	10	10	5	10
Primary endpoints	5 years; invasive IBE-free survival rate	10 years; invasive IBE-free survival rate	2 years: invasive IBE rate 5, 7 years: Invasive IBE rate	5 years; invasive IBE rate	invasive IBE-free survival rate
Secondary endpoints	n/s	Time to: IBE grade III DCIS, DCIS CBE, invasive CBE, treatment, distant metastases free interval, overall survival	2 years: MST/BCS rate, invasive CBE rate, overall and disease specific survival 5, 7 years: Overall and disease specific survival	5 years; invasive IBE rate, disease specific survival, invasive CBE-free survival rate, surgery rate, time to surgery, time-to-treatment failure, adverse events	Rate of invasive disease and rate of higher-grade DCIS in final specimen
Phase	III	III	III	III	III
Study arm 1	Surgery + AS	Surgery ± RT ± HT + AS	Surgery ± RT ± HT + AS	HT	Surgery ± RT + AS
Study arm 2	AS	AS	AS*	n/a	AS
Age	≧46	≧45	≧40	40–75	≧55
Grade	1 or 2	1	1 or 2	1 or 2	1 or 2
Size (cm)	n/s	Any size	n/s	≦2.5 cm	≦2.5 cm
Comedo necrosis	Excluded	n/s	Eligible	Excluded	Excluded
HR positivity	n/s	n/s	ER + and/or PR + and HER2-	ER + and HER2-	ER/PR + and HER2-
Biopsy method	VACB	VACB	VACB or Surgical biopsy	CNB or VACB	n/s
Imaging type	MMG	MMG	MMG	MMG, US and MRI	MMG
Imaging criteria	Screen detected by calcifications only	Screen detected by calcifications only	Screen detected by calcifications only	Screen detected by calcifications only	Screen detected by calcifications or tumor mass
Palpable mass	Excluded	Excluded	Excluded	Excluded	n/s
History of BC	Excluded	Excluded	Excluded	Excluded	n/s
Target cohort size	932	1240	1200	340	470

*IBE* Ipsilateral breast event, *CBE* Contralateral breast event, *MST* Mastectomy, *BCS* Breast conserving surgery, *AS* Active surveillance (annual mammogram), AS* Active surveillance (6 monthly mammogram), *RT* Radiotherapy, *HT* Hormone therapy, *ER* Estrogen receptor, *PR* Progesterone receptor, *HER2* Human epidermal growth factor receptor 2, *VACB* Vacuum assisted core biopsy, *CNB* Core needle biopsy, *MMG* Mammogram, *US* Ultrasound, *MRI* Magnetic resonance imaging, *BC* Breast cancer, *n/s* Not specified, *n/a* Not applicable

## Modelling the Transition from DCIS to IDC

**Fig. 2:**
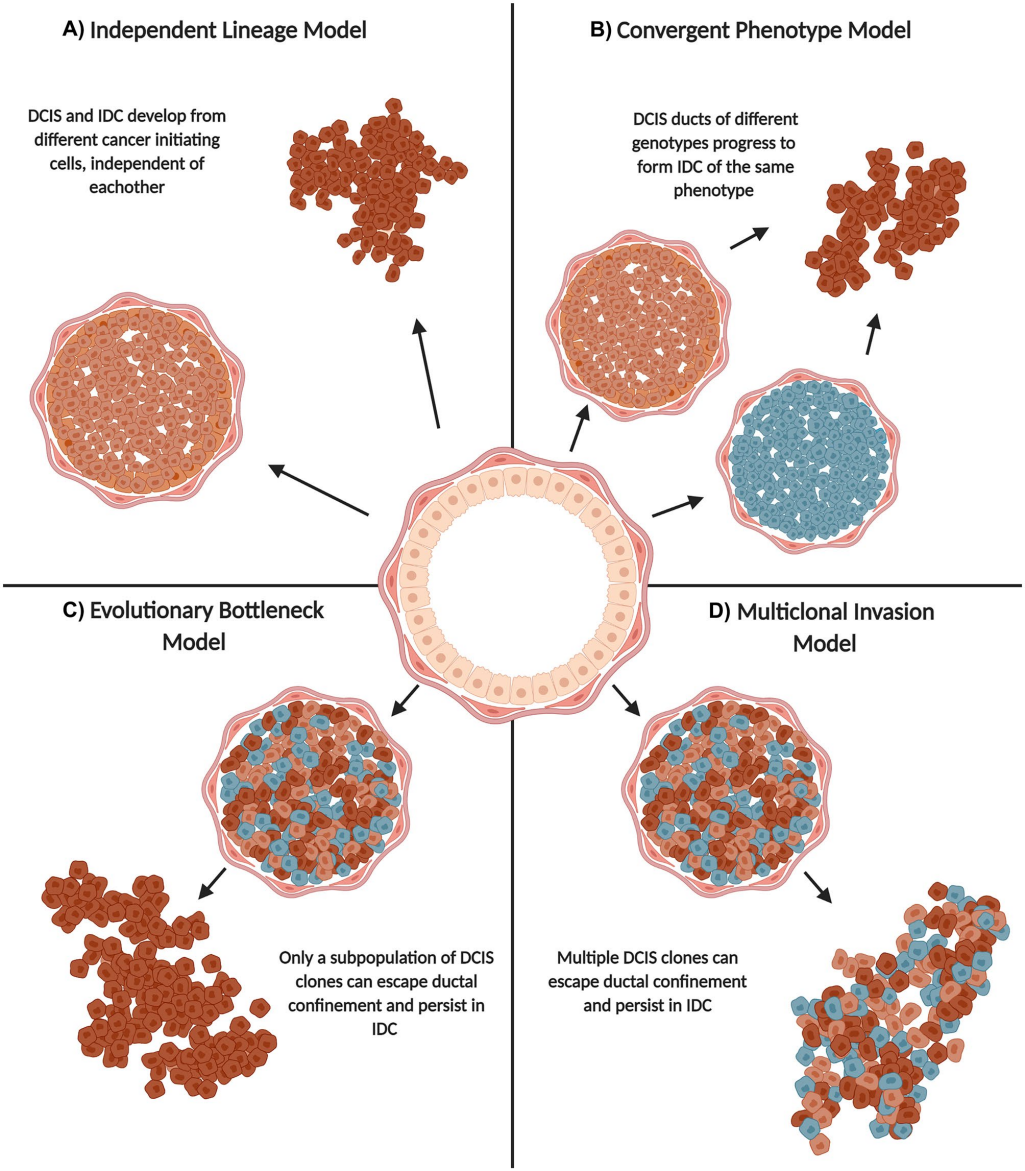
Models of ductal carcinoma in situ progression. Overview of the four theorized models of ductal carcinoma in situ progression. Drawing created with BioRender.com.

Phenotypically, the progression of DCIS to IDC is characterized by disruption of the myoepithelium and the basement membrane (Fig. 1) [43]. The breakdown of these layers facilitates tumor cell microinvasion and migration into the stroma, leading to invasive breast cancer [44]. While the key cellular mechanisms driving this progression are not fully defined, four models outlining the possible evolution have been proposed; the independent lineage model, the convergent phenotype model, the evolutionary bottleneck model and, the multiclonal invasion model (Fig. 2) [45]. While the independent lineage model theorizes that DCIS and IDC develop from different cancer initiating cells, independent from each other (Fig. 2A), the other models assume that DCIS and IDC develop from a common ancestor [46]. The convergent phenotype model posits that DCIS tumors with different genetic profiles progress to form invasive tumors of the same phenotype (Fig. 2B)[45]. The evolutionary bottleneck model proposes that while DCIS harbors neoplastic cells with different genetic profiles, only a single clone with a specific profile invades, migrates, and forms the invasive tumor (Fig. 2C) [45, 46]. In contrast, the multiclonal invasion model theorizes that multiple clones are able to invade, migrate and form the invasive tumor (Fig. 2D) [45, 46]. Genetic analyses of synchronous DCIS and IDC have demonstrated consistent mutational signatures between the invasive and non-invasive components [33, 47, 48], supporting the latter three evolutionary models over the former. In particular, the limited genomic changes that occur as the neoplastic cells progress from an in situ to invasive phenotype lends support to the multiclonal invasion model, since multiple genetically distinct clones that are present in the in situ tumor persist into the invasive carcinoma [48]. These results also highlight the very few intrinsic changes as DCIS cells progress from an in situ to invasive phenotype, suggesting the transition to invasive breast cancer is governed by extrinsic stimuli instead. In fact, studies of synchronous atypical ductal hyperplasia and DCIS show that mutations in key drivers, such as PIK3CA and p53, are already present in the pre-malignant state and very few additional mutational changes occur as early breast lesions progress along the malignant continuum [49]. Together, these findings suggest that all neoplastic cells can become invasive given sufficient time. The observation of intratumor grade heterogeneity in both DCIS and IDC further supports the multiclonal invasion model and opposes the convergent evolution model. Low grade DCIS and high grade DCIS are thought to evolve from two distinct evolutionary lineages, as each demonstrates distinct genomic signatures in the DCIS to IDC continuum [47, 50]. Whereas low grade DCIS progresses to well differentiated low grade IDC, high grade DCIS progresses to poorly differentiated high grade IDC [51]. Thus, clinical observations of heterogenous grade IDC are further evidence for the multiclonal invasion model, as multiple DCIS clones of both low and high grade must have progressed from heterogenous grade DCIS. Other investigations aiming to identify key changes that drive the progression of DCIS have compared the mutational landscape of pure and mixed DCIS lesions. Pure DCIS lesions are those that are diagnosed as in situ only whereas mixed DCIS lesions are those that are adjacent to invasive disease on diagnosis (synchronous lesions). Here, an assumption is that the IDC component was part of the adjacent DCIS before progression. These studies have reported a positive association between frequency of copy number alterations and DCIS progression [33, 52–54]. Moreover, one of the smaller studies reported a significantly lower frequency of driver mutations in pure DCIS lesions compared to mixed DCIS lesions and concluded that DCIS lesions with recurrent IDC show more aggressive genomic profiles than DCIS lesions with no recurrent IDC [33]. Combined, these results suggest there is potential to distinguish high and low risk DCIS at the genomic level, but more research is needed to accurately identify the defining genomic features. Overall, the genomic similarity between synchronous DCIS and IDC reveals that the DCIS-IDC transition is not governed by a single transformative change, nor is it dependent on a single DCIS clone of a certain genotype, as the evolutionary bottleneck model suggests. Instead, it is likely governed by small and successive changes in the tumor and/or stromal components, which together result in the invasive lesion.

## Experimental Models of DCIS Progression

Our understanding of DCIS and factors influencing its progression to invasive disease is limited by how it can be modelled for research. A handful of DCIS cell lines, mostly from cell derived xenografts, are available for analysis. Three-dimensional organoid cultures can be made using these cell lines to recapitulate DCIS structures that may show invasion [55], although such models do not encapsulate the true nature of DCIS progression in vivo. Moreover, most DCIS cell lines represent triple negative (MCF10DCIS.COM, S3 in the HMT-3522 series, h.DCIS.01 and SUM102PT) or ER-/PR-/human epidermal growth factor receptor 2 (HER2) + (21NT and SUM-225) DCIS [55], with limited representation of the more common ER + /PR + DCIS subtype. DCIS can be modelled in vivo via the mouse intraductal (MIND) transplantation model whereby tumor cell suspensions from cell lines or dissociated primary tumors are introduced and grown inside the duct [56]. This method is commonly employed to mimic the natural progression of non-invasive lesions, as all stages of the growth and progression of the tumors can be observed, as well as their interaction with the microenvironment [57]. Moreover, the MIND transplantation model allows for the growth of ER + DCIS structures without the need for exogenous estradiol, which had been notoriously difficult to establish through traditional mammary fat pad transplantation methods [57]. In fact, ER + MCF7 cells transplanted into the fat pad develop a basal-like molecular profile losing the expression of luminal markers whereas ER + MCF7 cells transplanted intraductally maintain their luminal profile [57]. Primary DCIS cells are superior to cell lines for modelling DCIS in vivo as they more closely embody the heterogeneity of the disease, although models are difficult to establish due to the insufficient amounts of primary tissue typically available for research purposes. Furthermore, human tumor cells grown in mice are not a true representation of human disease, since the model requires an immunosuppressed environment, which disregards the potential key role the immune system plays in DCIS progression. Early-stage lesions with the potential to progress to invasive cancer can be induced in rodents, rather than introduced, to model DCIS in vivo, and are referred to as mammary intraepithelial neoplasias (MINs) [58]. MINs used to model breast cancer progression have been induced by carcinogen exposure, irradiation and prolonged hormone treatment [59], as well as in genetically engineered mouse models in transgenic mice [60], knock out mice [61] or by viral transfection of oncogenic mutations [62]. The STAT1 knock out model, in which STAT1 deficient mice spontaneously develop ER + /PR + MINs, is a particularly useful model for studying the development of ER + breast cancer and suggests a critical role of the transcription factor in suppressing tumorigenesis [61, 63]. However, while inducing MINs in mice is a closer representation of DCIS progression in humans than xenografted cell lines, differences in the microenvironment in mice and humans, and the effects these have on tumor progression must be considered as a limitation of this approach. Investigators have sought to address this by generating mixed xenografts containing human DCIS, fibroblast, and myoepithelial cells, co-transplanted under the mouse kidney capsule [64, 65]. However, these models, while reproducing microenvironmental signaling, fail to maintain the normal topology of DCIS-affected ducts and, as in the MIND model, they lack immune context [64]. Notwithstanding their limitations, the above-mentioned models have proved very useful for mirroring the progression of non-invasive lesions like DCIS to invasive breast cancer in the laboratory setting, and further development, improvement and utilization of these models will likely provide a deeper understanding of the natural course of DCIS.

## Clinicopathologic Features Associated with DCIS Recurrence

Given the difficulty in predicting an individual patient’s risk of DCIS recurrence, there has been considerable interest in identifying specific clinicopathological features that have predictive potential. Numerous randomized controlled clinical trials and observational studies have investigated the correlations between various clinicopathologic features and DCIS recurrence. These study findings have been examined in four independent meta-analyses that have established composite correlations with overall recurrence (in situ or invasive) [66, 67] and invasive [68, 69] recurrence (Table 3). Among the four meta-analyses, a significant positive association was consistently reported between overall risk of recurrence and pre-menopausal status, young age, African-American race, symptomatic versus screen detected DCIS, presence of positive surgical margins, high grade, multifocality, presence of comedo necrosis, HER2 positivity and a family history of breast cancer [66–69]. Positive associations between overall recurrence and tumor size, or absence of ER and PR, have also been reported, although the data are either inconsistent or did not always reach significance [66, 67]. Only the presence of positive surgical margins showed a consistent positive association with invasive recurrence that was significant across the studies [68, 69]. Furthermore, given the high rate of ER and PR positivity in DCIS, hormone receptor negative DCIS cases are acutely under-represented, hindering comparison of recurrence rates between these groups and impacting the statistical strength of these studies. While clinical and histopathological features may be correlated with disease risk, their variability in prognostic accuracy means no features can be used in isolation to predict risk or guide management. One approach to improve the prognostic accuracy derived from clinicopathological data is to combine several independent features to formulate a single numerical index. One of these is the Van Nuys Prognostic Index (VNPI), which considers nuclear grade, tumor size, margin width, necrosis, and patient age, to develop a numerical score ranging from 4 (low likelihood of recurrence) to 12 (high likelihood of recurrence) [70]. In a random effects analysis of six studies that applied the VNPI, higher scores were consistently predictive of a substantially greater risk of an ipsilateral breast cancer recurrence. However, only one study found that a score of 10–12 was associated with an increased risk of mortality compared with a score of 4–6 [66]. While the prognostic utility of histopathological data may be enhanced when individual features are assessed in combination, interobserver variation in pathologic interpretation creates challenges for applying standardized risk-estimation systems, like VNPI. The lack of reliable, reproducible, and agreed-upon risk stratification methods that use clinicopathologic features, has inspired the consideration of genomic signatures and biomarkers to help reduce subjectivity and increase accuracy of prognostication.

**Table 3:** Association between clinicopathologic features and DCIS recurrence in four meta-analyses [66–69]

	**Association between clinical feature and overall IBE (DCIS or invasive)**		**Association between clinical feature and invasive IBE**	
	**Shamliyan et al. 2010 **[66]	**Wang et al. 2011 **[67]	**Zhang et al. 2016 **[68]	**Visser et al. 2019 **[69]
**Objective**	Association between clinical feature and overall clinical outcome (overall IBE and mortality)	Association between clinical feature and overall recurrence rates (DCIS and/or invasive)	Association between clinical feature and invasive breast cancer recurrence rates	Association between clinical feature and invasive breast cancer recurrence rates
**Inclusion criteria**	n/s	Minimum 100 cases per study (excluding biomarker analyses)	Minimum 100 cases per study, females only, HR, OR, or RR data with 95% CI, outcome data on ipsilateral invasive breast cancer	At least 1 year follow-up, more than 10 invasive recurrences in cohort
**Number of publications included**	133	37	18	40
**Number of RCT's**	5	5	5	5 RCT + 1 nRCT (non-randomized- ECOG trial E5194)
**Number of observational studies**	64	26	13	31
**Pre-menopausal status and/or age**	All studies consistently showed young age as a risk predictor of IBE, with one RCT finding women under 49 to have a 117% increased risk of IBE (HR = 2.17, 95% CI = 1.61 to 2.94). Similarly, premenopausal women are at significantly increased risk of invasive IBE compared to postmenopausal women	Not assessed	Not assessed	Premenopausal status significantly increased risk of invasive IBE (ES: 1.59: 95% CI: 1.20–2.11)
**Race**	Unadjusted results show African American DCIS patients have significantly increased mortality (RR = 1.35, 95% CI = 1.12 to 1.62) and invasive IBE (RR = 1.50 95% CI = 1.2 to 2) rates compared to Caucasian women	Not assessed	Not assessed	African American women have a significantly increased risk of invasive IBE (ES: 1.43: 95% CI: 1.15–1.79)
**Mode of detection**	RCT showed risk of IBE is significantly increased for symptomatic DCIS patients (RR = 1.9, 95% CI = 1.36 to 2.65) compared to patients detected by mammography	Women with palpable mass or other symptoms were significantly associated with an increased risk of IBE (HR = 1.35, 95% CI = 1.12–1.62)	A significant association between symptomatic (or non-screen detected) DCIS and increased risk of invasive IBE compared to screen detected DCIS (HR = 1.38, 95% CI = 1.12–1.63)	Detection by palpitation was significantly associated with increased risk of invasive IBE (ES: 1.84: 95% CI: 1.47–2.29)
**Patient BMI**	Overweight (RR = 2.3, 95% CI = 1.1 to 4.8) and obese (RR = 5.0, 95% CI = 1.1 to 10.8) patients are at significantly increased risk of invasive IBE	Not assessed	Not assessed	Not assessed
**Family history**	Risk of IBE was significantly associated with positive family history of breast cancer (HR = 3.08, 95% CI = 1.04 to 9.1)	Not assessed	Not assessed	Not assessed
**Mammographic breast density**	NSABB project B17 trial found that IBE rates are higher in women with higher breast density compared to women with lower breast density (RR = 2.8, 95% CI = 1.3–2.8)	Not assessed	Not assessed	Not assessed
**Tumor size**	Tumor size positively correlated with high risk of IBE, however not significant	Significant association between increased tumor size and increased risk of IBE (HR = 1.63 95% CI = 1.30–2.06) *	Inconsistent results among studies, no association	Not assessed
**Surgical margins**	Significant association between positive margins and increased risk of IBE. Margins greater or equal to 10 mm were associated with the largest reduction of risk (98%)	Significant association between positive margins and increased risk of IBE (HR = 2.25, 95% CI = 1.77–2.85) *	Significant association between positive margins and increased risk of invasive IBE (HR = 1.36, 95% CI = 1.04–1.69) I^2^ = 39.7	Significant association between positive margins and increased risk of invasive IBE (ES: 1.63: 95% CI: 1.14–2.32)
**Nuclear grade**	Significant association between high grade and increased risk of IBE (HH = 2.04, 95% CI = 1.63–2.56)	Significant association between high grade and increased risk of IBE (HR = 1.81, 95% CI = 1.53–2.13)	Non-significant association between high grade (HR = 1.04; 95% CI = 0.84–1.24) and increased risk of invasive IBE	Significant association between high grade and increased risk of invasive IBE (ES: 1.36: 95% CI: 1.04–1.77)
**Multifocality**	Not assessed	Significant association between multifocality and increased risk of IBE (HR = 1.95, 95%CI = 1.59–2.40)	Non-significant association between multifocality and increased risk of invasive IBE (HR = 1.34, 95% CI = 0.82–1.87)	Not assessed
**Comedo necrosis**	For lumpectomy patients, there is a significant association between comedonecrosis and increased risk of IBE (HR of 2.16, 95% CI = 1.26–3.69)	Significant association between comedonecrosis and increased risk of IBE (HR = 1.71, 95% CI = 1.36-.16) *	Non-significant association between comedonecrosis and increased risk of invasive IBE (HR = 1.18, 95% CI = 0.98–1.37) I^2^ = 4.2	Non-significant association between comedonecrosis and increased risk of invasive IBE (HR = 1.25, 95% CI = 0.98–1.6)
**ER positivity**	Non-significant association between ER positivity and reduced risk of IBE	Significant association between ER positivity and reduced risk of IBE (HR = 0.39, 95% CI = 0.18–0.86)	Non-significant association between ER positivity and decreased risk of invasive IBE (HR = 0.74), 95% CI = 0.36–1.12)	No association found
**PR positivity**	Not assessed	Non-significant association between PR positivity and reduced risk of IBE (HR = 0.56, 95% CI = 0.25–1.24)	Non-significant association between PR positivity and decreased risk of invasive IBE (HR = 0.89), 95% CI = 0.47–1.31)	Non-significant association between PR positivity and reduced risk of invasive IBE (HR = 0.80, 95% CI = 0.61–1.05)
**HER2 amplification**	Significant association between HER2 positivity and increased risk of IBE	Significant association between HER2 positivity and increased risk of IBE (HR = 3.07, 95% CI = 1.32, 7.12)	Non-significant association between HER2 positivity and increased risk of invasive IBE (HR = 1.25, 95% CI = 0.7–1.81)	Non-significant association between HER2 positivity and increased risk of invasive IBE (HR = 1.1, 95% CI = 0.75–1.62)
**Van Nuys prognostic index (VNPI)**	Women in higher risk categories had higher rates of IBE, the greatest increase was seen comparing women with VNPI scores of 5–7 to women with scores of 3–4, with HR of 8.4 (3.34–21.13)	Not assessed	Not assessed	Not assessed

*DCIS* Ductal carcinoma in situ, *IBE* Ipsilateral breast event, *n/s* Not specified, *HR* Hazard ratio, *RR* Risk ratio, *CI* Confidence interval, *ES* Pooled estimate, *RCT* Randomized controlled trial, *ER* Estrogen receptor, *PR* Progesterone receptor, *HER2* Human epidermal growth factor receptor 2, ***VNPI*** Van Nuys prognostic index

*Significant degree of heterogeneity between studies

## Molecular Signatures of DCIS

Breast cancer is recognized as a heterogeneous disease, and classification into clinically different subtypes is an important component of standard treatment. For IDC, subtype classification and estimation of risk of recurrence have traditionally been achieved by the assessment of a combination of clinical and pathological features, including tumor size, grade, node involvement, expression of ER and PR, and amplification of HER2. More recently, advances in high throughput molecular profiling approaches have demonstrated that breast cancers could be more accurately classified by considering the expression patterns of specific gene signatures. This finding has resulted in the development of several gene signature-based prognostic assays for clinical application [71]. Two commercially available molecular signature-based assays intended to guide the management of DCIS patients are outlined in Table 4. The Oncotype DX DCIS and DCISionRT predict the 10-year risk of local recurrence after BCS alone to assist clinicians’ decision making in recommending radiotherapy after surgery. The Oncotype DX DCIS assay uses reverse transcription quantitative PCR to measure the expression of a panel of seven genes, which were selected from the 21-gene Oncotype DX panel that predicts risk of recurrence in ER positive invasive breast cancer. Five signature development datasets, comprising only DCIS or DCIS and invasive breast cancer cases, were used to select the seven gene panel based on their association with recurrence. The panel was then independently validated using the ECOG E5194 study cohort [72]. The final signature comprises five genes involved in proliferation (Ki-67, STK15, Survivin, CCNB1 and MYBL2), plus progesterone receptor (PR) and the cancer susceptibility gene GSTM1 [72]. The normalized expression values are combined into a single numerical DCIS Score between 0 and 100, and results are stratified into low (DCIS Score < 39), intermediate (DCIS Score = 39–54) and high (DCIS Score ≥ 55) risk of recurrence. The DCISionRT test is an immunohistochemical assay that predicts risk of recurrence by measuring the expression of molecular biomarkers and clinicopathologic features, which were selected from a series of literature reviews. The protein biomarkers assess hormone response (PR, FOXA1), aggressiveness (HER2), proliferation (Ki-67), cell cycle regulation (p16/CDKN2A) and stress response (COX2, SIAH2), while the clinicopathologic features include age, tumor size, margin status and palpability [73] (Table 4). The elements of this biological signature were parameterized and cross-validated to develop the Decision Score (DS) ranging from 0–10, which stratifies patients into the low risk (DS ≤ 3) or high risk (DS > 4) group. These signatures predict risk with some degree of accuracy, but validations have revealed that they tend to overtreat a proportion of cases yet still miss recurrence in the putative low risk group [72, 74–77]. A validation of the Oncotype DX DCIS assay found that 12.7% of patients classified as low risk showed some kind of recurrence within 10 years and 72.2% of patients classified as high risk, who were recommended more aggressive treatments showed no recurrence within 10 years [77]. Moreover, the test is of uncertain value in cases that return an intermediate score, which are arguably the cohort most in need of enhanced guidance. Validation of the DCISionRT test revealed that 10% of the low risk patients will show recurrence within 10 years and 70% of the high risk patients will not show any recurrence within 10 years [74]. Overall, these tests provide some guidance, but improvements in their accuracy would allow more patients to be spared overtreatment. Still lacking however, is a means of predicting DCIS progression. Development of an assay that can accurately identify those cases that are not predicted to progress to invasive disease would negate even surgical intervention for a significant proportion of patients. Each of the tools above focuses exclusively on the malignant component of DCIS. Given the similar molecular profiles of DCIS and IDC, it is likely that other factors related to the adjacent microenvironment and myoepithelial component of DCIS should be considered when attempting to predict the risk of recurrence in these in situ lesions.

**Table 4:** Features of the Oncotype-DX DCIS and DCISionRT commercial tests

	**Oncotype-DX DCIS** [72]	**DCISionRT** [73]
Cost	~ $3000 USD	~ $1500 USD
Type of molecular signature	Gene expression via RT-PCR	Biomarker expression via IHC coupled with clinical features
Genes/markers/clinical features	Ki-67, STK15, Survivin, CCNB1, MYBL2, PR, GSTM1 (cancer related genes) & ACTB, GAPDH, RPLPO, GUS, TFRC (housekeeping genes)	COX2, FOXA1, HER2, Ki-67, p16/CDKN2A, PR, SIAH2 (IHC) and patient age, tumor size, margin status and palpability
Range of score	**0–100**	**0–10**
Scoring risk of local recurrence	Low risk score < 39 Intermediate risk score = 39–54 High risk score $\ge$ 55	low risk score < 3 high risk score > 3

## Molecular Changes Accompanying the DCIS-IDC Transition

### Molecular Changes to the DCIS Cells

As DCIS cells evade ductal confinement, they undergo a number of phenotypic changes. Invasion and migration through the stroma is aided by epithelial to mesenchymal transition (EMT), where polarized epithelial cells de-differentiate, biochemically changing to a mesenchymal phenotype [78]. Compared to DCIS cells, IDC cells show significantly increased expression of putative EMT markers c-MET and TGF-ß1 [79]. Further research is needed to deduce whether phenotypic changes related to EMT are present as early as the DCIS stage for those whose DCIS progressed to IDC, as early expression changes may be useful for identifying DCIS patients at high risk of invasive progression. Invasion through the stroma is also aided by ECM remodeling, where components of the ECM become deposited, modified or degraded [80]. Recent evidence suggests that DCIS cells upregulate the expression of ECM remodeling proteases which aids in invading the stroma. DCIS cells adjacent to invasive disease show significantly higher expression of ECM remodeling proteases cathepsin V and cathepsin A, prolyl 4-hydroxylase A2 (involved in collagen synthesis) and fibrillar collagen component COL11A1, compared to DCIS cells from pure lesions (Table 5), suggesting the expression of these proteins aids in the DCIS-IDC transition. Furthermore, the tumor cells that have evaded ductal confinement show significantly higher expression of cathepsin V, cathepsin A and COL11A1 compared to their adjacent non-invasive component, suggesting these proteins play a significant role in DCIS progression (Table 5). Studies have also shown that as DCIS progresses to IDC, DCIS cells gradually lose expression of growth suppressive protein thioredoxin interacting protein and gradually gain expression of proliferation protein legumain [81, 82]. Importantly, these phenotypic changes are visible in the pre-invasive state, where DCIS lesions adjacent to invasive disease show significantly lower expression of thioredoxin interacting protein but significantly higher expression of legumain, compared to pure DCIS lesions (Table 5). Given the immune system is capable of detecting and eliminating tumor cells, tumor cells induce changes to modulate the immune system [78]. DCIS cells express immune regulatory protein PD-L1 which binds to PD-1 receptors on activated T cells to inactivate them [83–85]. It is likely that there are other immune modulating mechanisms employed by DCIS cells to evade immune surveillance, although these are yet to be uncovered. Such mechanisms may also serve as valuable targets for therapeutics.

**Table 5:** Molecular changes in DCIS (pure and mixed) and IDC tissue and their prognostic significance

**Marker**	**Biological mechanism(s)**	**Expression in normal tissue**	**Expression in DCIS compared to normal tissue**	**Expression in pure and mixed DCIS tissue**	**Expression in IDC compared to synchronous DCIS**	**Association with recurrence**	**Association with clinical features**	**Reference**
VAV2 in tumor cells	Activates the Rho family of GTPases and induces EMT	Cell membrane of normal epithelial cells	Similar expression in DCIS compared to normal (*P* = 0.11)	Significantly higher expression in mixed DCIS lesions compared to pure (*P* = 0.03)	Not assessed	Not assessed	Not assessed	Jiang et al. 2014 [126]
Thioredoxin interacting protein (TXNIP) in tumor cells	Negative regulator of antioxidant thioredoxin with growth suppressing and pro-apoptotic functions	Cytoplasmic expression in luminal and myoepithelial cells	Reduced expression in DCIS tumor cells compared to normal epithelial cells	Significantly higher expression in pure DCIS cells compared to mixed DCIS cells (*P* < 0.0001)	Significantly higher expression in DCIS cells compared to adjacent IDC cells (*P* < 0.0001)	High expression is associated with longer local recurrence-free survival (*P* = 0.009)	High expression is associated with low grade (*P* = 1.6 × 10^–5^), absence of comedo necrosis (*P* = 0.001), ER positivity (*P* = 2 × 10^–6^) and HER2 negativity (*P* = 0.007)	Miligy et al. 2018 [81]
Legumain in fibroblasts	Proteolytic enzyme that regulates cell proliferation	Negative or faint expression in normal fibroblasts	Cytoplasmic expression in DCIS fibroblasts	Significantly more mixed DCIS cases showed high fibroblast expression compared to pure DCIS (*P* < 0.0001)	Significantly higher expression in fibroblasts of IDC compared to fibroblasts of the adjacent DCIS (*P* < 0.0001)	Not associated with recurrence	High expression in fibroblasts of pure DCIS tissue is associated with high grade (*P* = 0.002), comedo necrosis (*P* = 0.033), ER negativity (*P* < 0.0001), PR negativity(*P* < 0.0001), HER2 positivity (*P* = 0.01), high proliferative index (*P* < 0.0001) and dense tumor-infiltrating lymphocytes (*P* < 0.0001)	Toss et al. 2019 [82]
Legumain in tumor cells	Proteolytic enzyme that regulates cell proliferation	Negative or faint expression in epithelial cells	Cytoplasmic staining of DCIS tumor cells	Significantly more mixed DCIS cases showed high tumor cell expression compared to pure (*P* = 0.001)	Significantly higher expression of legumain in the IDC tumor cells compared to the adjacent DCIS cells (*P* < 0.0001)	High tumor cell expression associated with shorter LRFI (*P* = 0.0002)	High tumor cell expression in pure DCIS is associated with high grade (*P* < 0.0001), comedo necrosis (*P* = 0.012), ER negativity (*P* < 0.0001), PR negativity(*P* < 0.0001), HER2 positivity (*P* = 0.002), high proliferative index (*P* = 0.001) and dense tumor-infiltrating lymphocytes (*P* = 0.002)	Toss et al. 2019 [82]
Cathepsin V in fibroblasts	Lysosomal cysteine proteinase involved in ECM degradation	Negative or faint expression in fibroblasts	20% of pure DCIS cases had high stromal CTSV	Significantly more mixed DCIS cases showed high stromal expression of CTSV compared to pure (*P* = 0.001)	Significantly higher expression in stroma of IDC compared to adjacent DCIS (*P* < 0.0001)	High stromal expression is associated with high rate of all ipsilateral recurrences (*P* = 0.001) and invasive recurrences (*P* = 0.008)	High stromal cell CTSV associated with larger tumor size (*P* = 0,025), high nuclear grade (*P* < 0.0001), comedo necrosis (*P* = 0.003), ER negativity (*P* < 0.0001), PR negativity (*P* < 0.0001), HER2 positivity (*P* < 0.0001), High Ki-67 (*P* = 0.0001), dense TIL infiltrate (*P* = 0.002), high HIF1alpha expression (*P* < 0.0001)	Toss et al. 2020 [97]
Cathepsin V in tumor cells	Lysosomal cysteine proteinase involved in ECM degradation	Negative or faint expression in epithelial cells	29% of pure DCIS cases had high tumor cell CTSV	Significantly more mixed DCIS cases showed high tumor cell expression of CTSV compared to pure (*P* < 0.0001)	Significantly higher expression in IDC tumor cells compared to adjacent DCIS cells (*P* < 0.0001)	High tumor cell expression is associated with shorter LRFI for pure DCIS patients (*P* = 0.013)	High tumor cell expression is associated with high grade (*P* = 0.021), ER negativity (*P* < 0.0001), PR negativity (*P *= 0.006), HER2 positivity (*P* = 0.001) and high HIF1alpha expression (*P* = 0.042)	Toss et al. 2020 [97]
Cathepsin A in tumor cells	Lysosomal serine proteinase involved in ECM remodeling, resistance to apoptosis, cell proliferation	Negative or faint expression in epithelial cells	Increased expression in DCIS tumor cells	Higher proportion of mixed DCIS cases show high expression compared to pure DCIS cases (*P *= 0.04)	Significantly higher expression in IDC tumor cells compared to the adjacent DCIS cells (*P *< 0.0001)	Higher CTSA expression is associated with shorter LRFI (*P* = 0.0001)	High tumor cell expression is associated with high grade (*P* = 0.006), ER negativity (*P* = 0.001), PR negativity (*P* = 0.011), HER2 positivity (*P* < 0.0001), high HIF1alpha (*P* = 0.002)	Toss et al. 2019 [96]
Prolyl-4-hydroxylase A subunit 2 (P4HA2) in tumor cells	Enzyme for ECM remodeling	Negative or faint expression in epithelial cells		Significantly higher proportion of mixed DCIS cases show high tumor cell expression compared to pure DCIS cases (*P* = 0.003)	No expression difference between IDC tumor cells and adjacent DCIS cells (*P* = 0.188)	High tumor cell expression is associated with ipsilateral local recurrence (*P* = 0.004)	High tumor cell expression is associated with older age (*P* = 0.004), high nuclear grade(*P* < 0.0001), comedo necrosis(*P* < 0.0001), ER negativity(*P* < 0.0001), PR negativity (*P* < 0.0001), HER2 positivity (*P* = 0.004), high HIF1-alpha expression (*P* < 0.0001)	Toss et al. 2018 [98]
Prolyl-4-hydroxylase A subunit 2 (P4HA2) in fibroblasts	Enzyme for ECM remodeling			Significantly higher stromal expression in mixed DCIS tissue compared to pure DCIS tissue (*P* < 0.0001)	Significantly higher stromal expression in IDC compared to adjacent DCIS (*P* < 0.0001)	Not associated with recurrence	High stromal expression is associated with symptomatic DCIS (*P* = 0.02), high grade (*P* = 0.04), comedo necrosis (*P* = 0.001), ER negativity (*P* = 0.001), PR negativity (*P* = 0.003), high HIF1-alpha expression (*P* = 0.004)	Toss et al. 2018 [98]
COL11A1 in fibroblasts	Component of collagen for ECM remodeling	Low expression in fibroblasts	High expression in DCIS fibroblasts	Significantly higher stromal expression in mixed DCIS compared to pure DCIS (*P* < 0.0001)	Significantly higher stromal expression in IDC compared to adjacent DCIS (*P* < 0.0001)	High expression is an independent predictor of shorter LRFI (*P* < 0.0001)	High stromal expression is associated with ER negativity (*P* = 0.002), PR negativity (*P* = 0.042), Ki-67 positivity (*P* = 0.049) dense tumor infiltrating lymphocytes (*P* = 0.016) and hypoxia-inducible factor 1 alpha (*P* < 0.0001)	Toss et al. 2019 [99]
COL11A1 in tumor cells	Component of collagen for ECM remodeling	Negative expression in epithelial cells	High expression in DCIS cells	Significantly higher expression in mixed DCIS tumor cells compared to pure DCIS tumor cells (*P* < 0.0001)	Significantly higher expression in IDC tumor cells compared to adjacent DCIS cells (*P* < 0.0001)	High tumor cell expression is associated with shorter LRFI for all recurrences (*P* = 0.001)	Low expression is associated with PR positivity (*P* = 0.012)	Toss et al. 2019 [99]
MMP8 in myoepithelial cells	Tumor suppressive MMP8 which opposes MMP9 tumor promoting activity and downregulates TGF-beta in myoepithelial cells	High expression in myoepithelial cells	45% and 81% of pure DCIS and mixed DCIS respectively, were negative for MMP8	MMP8 expression was significantly reduced in mixed DCIS tissue compared to pure DCIS tissue (*P* = 0.001)	No myoepithelial cells in IDC	No recurrence data		Sarper et al. 2017 [94]
Stefin A in myoepithelial cells	Protease inhibitor to inhibit ECM remodeling	High expression in myoepithelial cells	Myoepithelial cells from low grade DCIS showed similar expression to normal, but intermediate grade (*P* < 0.01) and high grade (*P* < 0.0001) DCIS tissue showed significant loss of Stefin A expression	Myoepithelial cells from DCIS tumors with microinvasion did not express any Stefin A	No myoepithelial cells in IDC	No recurrence data		Duivenvoorden et al. 2017 [95]
Integrin αvβ6 in myoepithelial cells	Receptor for ECM remodeling proteins	Negative expression in myoepithelial cells	69% of high grade and 52% of non-high-grade DCIS cases showed myoepithelial expression of αvβ6 compared to 0% expression in normal tissue	A significantly higher proportion of mixed DCIS cases showed positive expression of αvβ6 compared to pure DCIS cases (*P* < 0.001)	No myoepithelial cells in IDC	Significant association between high αvβ6 expression and recurrence or progression (*P* = 0.006) When adjusted for grade and size (*P* = 0.02)		Allen et al. 2014 [93]

*LFRI* Local recurrence free interval, *DCIS* Ductal carcinoma in situ, *EMT* Epithelial to mesenchymal transition, *ER* Estrogen receptor, *HER2* Human epidermal growth factor receptor 2, *CTSV* Cathepsin V, *CTSA* Cathepsin A, *P4HA2* Prolyl-4-hydroxylase A subunit 2

### Molecular Changes to the Adjacent Microenvironment

**Fig. 3:**
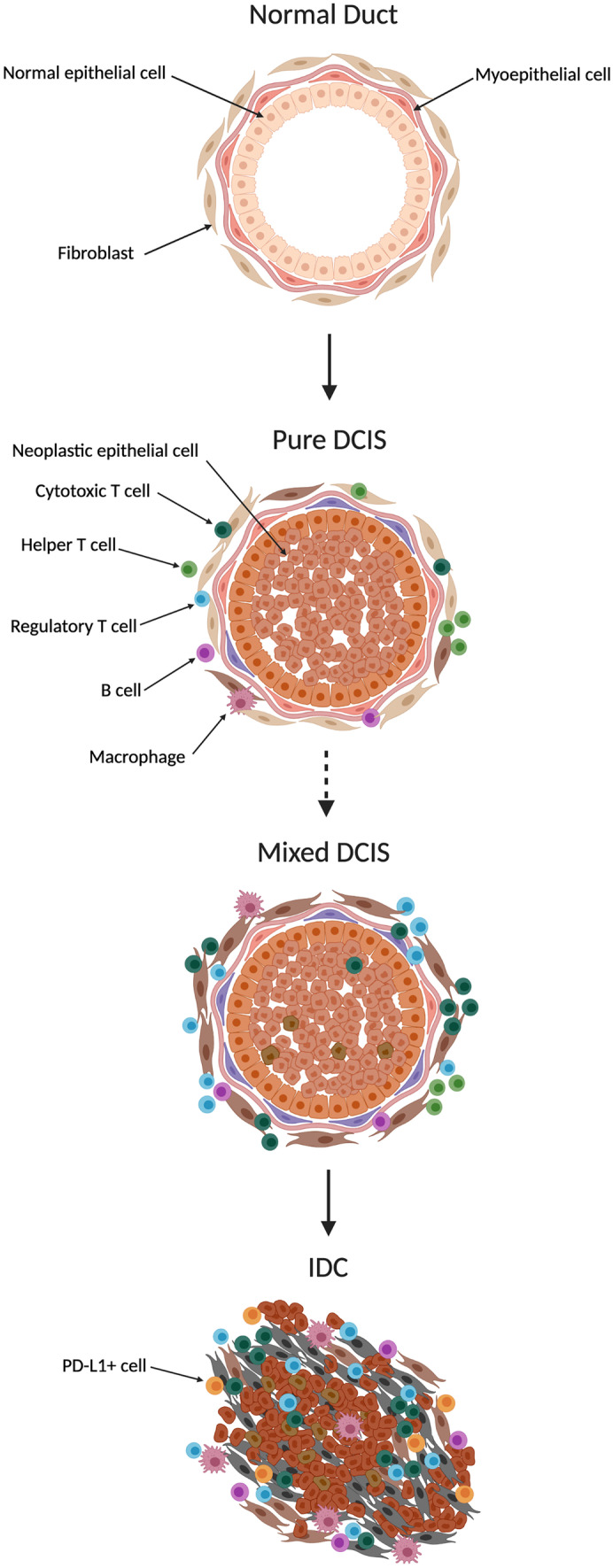
Microenvironment changes accompanying ductal carcinoma in situ progression. Changes to the microenvironment as normal tissue becomes progressively more neoplastic, along the ductal carcinoma in situ to invasive ductal carcinoma continuum. Development of fully confined “pure ductal carcinoma in situ” lesions are accompanied by measurable changes to the microenvironment, including myoepithelial and stromal alterations and increased immune infiltrate. These changes are progressively more distorted in “mixed ductal carcinoma in situ” lesions adjacent to invasive ductal carcinoma. Finally, development of invasive ductal carcinoma is accompanied by total loss of the myoepithelium, further stromal alterations and an immunosuppressive immune phenotype. Drawing created with BioRender.com.

Given that the majority of genetic changes occur early in the development of DCIS, the transition to invasive disease might not be regulated entirely at the genomic level but instead may be influenced by cellular relationships and interactions within and between DCIS and its microenvironment [86]. Emerging evidence demonstrates that cells of the DCIS ecosystem progressively undergo changes as DCIS becomes IDC (Fig. 3) and that some of these changes are prognostic when they are present in the pre-invasive state (Table 5). Understanding these changes to the DCIS ecosystem will yield new avenues to predict and prevent the progression to invasive breast cancer. The DCIS to IDC transition is characterized phenotypically by the fragmentation of the myoepithelium and the basement membrane. Loss of these physical barriers allows tumor cell invasion into the microenvironment and lymphovascular space. Normal myoepithelial cells perform several tumor-suppressive roles and, as a result, are often regarded as the “gatekeepers” of DCIS. However, before the myoepithelial cell layer is disrupted, DCIS-associated myoepithelial cells exhibit changes in the expression of structural proteins such as calponin, CD10 and smooth muscle myosin which normal myoepithelial cells express in abundance [50, 86–91]. Loss of structural proteins may compromise the actin cytoskeleton of the myoepithelium, affecting cellular integrity and diminishing the effectiveness of the myoepithelial barrier. Moreover, myoepithelial cells from DCIS lesions that have progressed to IDC show more aberrations than myoepithelial cells from DCIS lesions that have not progressed [50, 86, 88, 92], providing evidence for the idea that changes in the structural composition of myoepithelial cells can result in increased permeability for tumor cells to disseminate [92]. Myoepithelial cells also exhibit phenotypic changes in proteins involved in tumor progression. Myoepithelial cells surrounding DCIS lesions adjacent to invasive breast cancer show increased expression of proteins that promote ECM remodeling [93] and decreased expression of proteins that inhibit ECM remodeling [94, 95], compared to myoepithelial cells surrounding pure DCIS lesions that do not have adjacent invasive disease (Table 5). Similarly, mammary fibroblasts become progressively altered during malignant progression, showing increased expression of ECM remodeling proteins COL11A1, prolyl 4-hydroxylase A2 and cathepsin V, in DCIS-associated fibroblasts compared to normal tissue, and further increased expression in fibroblasts adjacent to IDC (Table 5) [82, 96–99], suggesting that these proteins contribute to a microenvironment favorable for breast cancer progression. Moreover, fibroblasts from DCIS lesions adjacent to invasive breast cancer show significantly higher levels of these proteins compared to fibroblasts surrounding pure DCIS lesions, demonstrating that the increased expression commencing in the pre-invasive state could be prognostic (Table 5) [82, 96–99]. Evidence also suggests that the transition to IDC is accompanied by signaling changes to aid cell migration. Compared to normal tissue, gene expression analysis of DCIS tissue revealed the RNA expression of chemokines CXCL14 and CXCL12 is elevated in myoepithelial cells and fibroblasts, respectively, both of which directly bind to receptors on the tumor cells to induce cell migration [100]. Additionally, CXCL12 recruits regulatory T cells to the tumor site, which act to suppress the immune response [101]. Moreover, the expression of CXCR4, the CXCL12 receptor is elevated in invasive tumors compared to both DCIS and normal tissue, lending support to the idea that increased chemokine signaling contributes to DCIS progression [100]. There is no evidence that the changes exhibited in the DCIS microenvironment are driven by mutations in the tumor adjacent stromal cells. Rather, it is likely that the cells of the DCIS microenvironment are manipulated by the tumor cells to facilitate DCIS progression. As the tumor cells proliferate and the myoepithelial barrier between tumor and stroma is progressively lost, there is more opportunity for the aberrant expression of proteases, chemokines, growth factors and remodeling molecules originating from the DCIS cells to stimulate a pro-tumor phenotype in the adjacent fibroblasts. While a single driving mechanism for the progression of DCIS is yet to be revealed, changes to the tumor cells and other neighboring cell types during the invasive transition may hold prognostic potential for predicting whether future DCIS patients are at high or low risk of invasive progression.

## Immune Populations in DCIS

Immune modulation is a critical hallmark of tumor progression, although until recently, breast cancer has largely been considered an immunologically silent cancer. That is, the immune infiltrate in breast tumor microenvironments was not used to classify the tumor or guide patient treatment. However, recent evidence proves that breast cancer can be highly immunogenic, containing immune cells from both the innate and adaptive immune system [101]. Immune cells within the breast cancer microenvironment shape tumor progression and treatment response, and can be stratified into two classes: immunostimulatory and immunosuppressive [101]. Immunostimulatory immune cells such as M1 macrophages, lymphocytes and natural killer (NK) cells play critical roles in tumor cell elimination whereas immunosuppressive immune cells such as myeloid derived suppressor cells (MDSCs), M2 macrophages, T regulatory cells (Tregs) and PD-L1+ immune cells inhibit the tumor suppressing functions of immunostimulatory immune cells, leading to tumor progression [101]. This section will discuss immune infiltrate changes that occur in the continuum of normal to DCIS to IDC breast tissue, including the associations that immune cell infiltrates have with clinicopathologic features and outcome in DCIS patients.

## Immune Changes in DCIS and IDC Tissue

The progression from normal to neoplastic breast tissue is accompanied by quantitative, compositional, and phenotypic changes to the immune cell infiltrate. Immunogenicity progressively increases as normal breast tissue becomes more neoplastic (Fig. 3). There is considerably more known about the immune environment of IDC than DCIS, however a few studies have sought to document the changes that occur in the transition from normal to DCIS to IDC tissue. There is a significantly higher proportion of CD3+ T cells, CD20 + B cells, macrophages and Tregs [102, 103] in DCIS tissue compared to adjacent normal tissue, while IDC tissue contains a significantly higher proportion of all T cells (helper, cytotoxic and regulatory), macrophages, B cells and PD-L1+ immune cells compared to adjacent DCIS tissue [102–104]. While helper T cells are more abundant than CD8+ cytotoxic T cells in DCIS tissue [85, 104–106], cytotoxic T cells are more abundant than helper T cells in IDC tissue [104], suggesting a more immune activated environment in IDC. Breast cancer cells can also stimulate the recruitment and differentiation of myeloid progenitor cells into MDSCs to facilitate immune evasion. Tumor cells have been reported to produce a range of soluble factors to attract and differentiate this immature immune subset, including granulocyte and macrophage colony stimulating factors (G-CSF and M-CSF), interleukin 6 and TGF-ß1 [107]. MDSCs accumulate aberrantly in cancers, diverting myeloid progenitors away from their normal differentiation pathway to dendritic cells, macrophages and granulocytes, towards a pathological state, suppressing CD8+ T cell function by the production of reactive oxygen species, and expression of the enzymes ARG1 and inducible nitric oxide synthase (iNOS) [108, 109] and promoting metastasis and osteolysis [107]. There is limited information about the relative abundance of MDSCs in DCIS compared to IDC. However, a murine model of breast cancer progression demonstrated increasing numbers of this immunosuppressive cell population with increasing tumor burden, and this was correlated with a decrease in CD8+ T cells [110], suggesting their increasing presence in DCIS could promote progression to invasive disease.

The immune infiltrate of DCIS tissue is predominantly seen in the stroma and is less concentrated in the transformed ducts themselves. Recent reports suggest that early immune evasion by the DCIS cells is likely aided by an intact myoepithelium, where one study reported that DCIS regions with disturbed myoepithelial cell phenotypes display T cell infiltrations within the duct whereas in areas with normal myoepithelial cell phenotypes the T cells remain in the surrounding stroma [92]. A recent spatial profiling study of the tumor microenvironment of DCIS from patients who remained free of invasive disease compared to tumors from patients who had developed subsequent IDC found that myoepithelial disruption was greater in cases that did not progress [111]. In that study, analysis of spatial relationships within the tumor microenvironment suggested that this disruption was associated with increased numbers of immune cells, cancer-associated fibroblasts and collagen remodeling, which combined to protect against future invasive relapse. However, the study did not report a difference in the immune cell type composition between progressors and non-progressors, and it remains to be seen whether the elevated immune presence associated with myoepithelial disruption was a consequence of or contributor to that phenotype. In contrast to this, in a study of malignant progression that employed a HER2 over-expressing mouse mammary tumor virus model, CD206+ /Tie2+ macrophages were observed to be increasingly present during the transition from normal ducts to in situ malignancy and invasive disease and contributed to cancer dissemination [112]. Depletion of macrophages during the premalignant stage of tumor development reduced subsequent dissemination, suggesting a direct causal role of the macrophages in promoting invasion and metastasis. Gene analysis performed by one group found that DCIS and IDC tissue were significantly more enriched for genes relating to NK cells than normal breast tissue, although they found no significant difference between DCIS and IDC tissue [85]. Overall, consistent evidence has demonstrated an increased immune cell presence in neoplastic breast tissue compared to normal tissue [85, 102–104].

## Clinical Significance of Immune Infiltrate in DCIS

An increased immune presence in DCIS tissue is associated with poor prognostic features including high grade, hormone receptor negativity, HER2 positivity and comedo necrosis, suggesting that more aggressive tumors trigger a more intense immune response than less aggressive tumors (Table 6) [84, 85, 104, 113–119]. Moreover, a stromal increase in any T cell subset, PD-L1+ immune cells, B cells and macrophages, is independently associated with aggressive clinical features [84, 85, 104, 113–119]. These trends are reflected in a correlation between immune cell content and DCIS molecular subtype. A 1488 patient study reported that the highest tumor infiltrating lymphocyte density was observed in HER2 + DCIS, followed by triple negative, luminal B/HER2+ and finally Luminal A/B [120]. Immune infiltrates are reportedly higher in mixed DCIS lesions compared to pure DCIS lesions (Table 6) [105, 114, 119] suggesting an increased immune response contributes to, or is perhaps predictive of, progression of DCIS to invasive disease. Similarly, a high abundance of immune cells in DCIS has been associated with an increased risk of local recurrence in a number of studies (Table 6) [84, 105, 114, 119]. Unsurprisingly, a high abundance of Tregs in DCIS tissue is associated with an increased risk of recurrence [104, 114, 121] and invasive progression [104, 114]. Studies also reported that mixed DCIS lesions contained significantly higher proportions of cytotoxic T cells compared to pure lesions, although this observation is likely confounded by the presence of Tregs and PD-L1+ immune cells which act to inhibit the tumor suppressing action of cytotoxic T cells (Table 6). It is likely that a higher level of infiltration of cytotoxic T cells alone would be associated with a lower risk of DCIS progression, given the anti-tumorigenic roles these cells play, and consistent with two separate studies that reported that low cytotoxic T cell infiltrates are associated with a high risk of recurrence (Table 6) [113, 115]. There is limited information on the specific macrophage populations in DCIS. Three studies have examined macrophage levels in DCIS (Table 6) and although higher macrophage levels were associated with higher grade, more aggressive disease, none distinguished macrophage subtypes, so whether the M1 or M2 type was preferentially elevated was not explored. The presence of MDSCs is another factor to consider when assessing the clinical significance of T cell infiltrates in DCIS, given their known role in promoting immune evasion by inducing T cell dysfunction and secreting immunosuppressive IL-10 [108]. To date, just one study has considered MDSCs in DCIS, but found no association between the presence of MDSCs and the fraction of activated T cells and suggested other mechanisms underlie the inactivated T cell infiltrate [85]. Further research into the role that MDSCs may play in promoting DCIS progression would benefit the field. In conclusion, an elevated immune presence, particularly an increase in Tregs or other immunosuppressive cells, in the DCIS microenvironment, is associated with poor patient outcome. These observations highlight the potential utility of predicting DCIS outcome using immune markers. However, for now, with limited studies available and considerable discordance between them, there is no consensus on the prognostic significance of immune cell quantity, composition, or location in DCIS tissue. Moreover, the consensus approach to clinical assessment of immune infiltrate requires evaluation of fixed tissue sections that are only available after surgical excision of the lesion [122]. It is possible that similar assessment could be conducted on core biopsy specimens, since research studies have achieved sufficient coverage using similarly small lesion areas from tissue microarrays [105]. However, further investigation would be required to demonstrate the robustness of such assessment to support clinical decisions. Further investigation into how the complex interactions between DCIS cells and the immune infiltrate contribute to DCIS outcome is warranted.

**Table 6:** Immune cell infiltrate in pure and mixed DCIS tissue and association with clinical features and DCIS outcome

Cell type	Immune cell in pure DCIS vs mixed DCIS (including microinvasion)	Immune cell association with clinical features	Immune cell association with outcome	Cohort details	Reference
**Overall immune infiltrate (all markers or CD45 +)**	Not assessed	High infiltrate of CD4+ , CD8+ and FOXP3+ TILs and the presence of PD-L1+ immune cells were associated with high nuclear grade (*P* < 0.05), comedonecrosis (*P* < 0.05), ER negativity (*P* < 0.05), PR negativity (*P* < 0.05) and high Ki-67 index (*P* < 0.05)	Not assessed	Pure DCIS (n = 231) & Mixed DCIS (n = 81)	Kim et al. 2020 [104]
	Mixed DCIS tissue had significantly more TILs compared to pure DCIS (*P* = 0.009)	ER- DCIS contained higher numbers of TILs compared to ER+ DCIS	DCIS lesions which recurred (DCIS or invasive) had higher numbers of TILs compared to DCIS lesions that did not recur	Pure DCIS (n = 24) & Mixed DCIS (n = 3)	Thompson et al. 2016 [105]
	Mixed DCIS lesions had significantly fewer immune cells (CD45) compared with pure DCIS lesions (*P* < 0.001), but a significantly higher proportion of CD3+, CD8+, CD8+/PD-1+ cells of all immune cells, compared to pure DCIS tissue	Not assessed	No outcome data	Pure DCIS (n = 19) & Mixed DCIS (n = 11)	Mitchell et al. 2020 [92]
	Higher density of TILs in mixed DCIS tissue compared to pure DCIS lesions	Increased TIL density was significantly associated with high Ki-67 index, ER negativity, PR negativity, p53, HIF1alpha	High TIL density was associated with all recurrences (*P* = 0.0001) and invasive recurrences (*P* = 0.04)	Pure DCIS (n = 508) & Mixed DCIS (n = 192)	Toss et al. 2020 [114]
	High density of TILs in mixed DCIS tissue compared to pure DCIS (*P* = 8.96 × 10^–13^)	Dense TILs are associated with younger age (*P* = 0.043), symptomatic presentation (*P* = 0.049), larger size (*P* = 0.001), higher nuclear grade (*P* = 1.1 × 10^–9^), comedonecrosis (*P* = 0.00009), ER negativity (*P* = 1.04 × 10^–10)^ and Paget's Disease (*P* = 0.025)	Univariate analysis: dense TILs are associated with shorter RFI for all patients (*P* = 0.005) and patients treated with BCS alone (*P* = 0.001). Multivariate analysis: dense TILs are associated with shorter RFI for BCS patients (*P *= 0.002)	Pure DCIS (n = 534) & mixed DCIS (n = 132)	Toss et al. 2018 [119]
	Not assessed	TIL density was significantly higher in high grade (*P* = 0.002), ER- (*P* = 0.002), HER2 + (*P* < 0.0001) and comedonecrosis (*P* < 0.0001) DCIS. High TIL density was associated with TP53 gene mutation (*P* = 0.033) and HER2 gene amplification (*P* = 0.001)	No association	Pure DCIS only (n = 138)	Hendry et al. 2017 [127]
	Not assessed	High grade DCIS had significantly higher TIL infiltrate (*P* < 0.01) compared to non-high grade DCIS. High TIL density was associated with high VNPI (*P* < 0.05), increased tumor size *P* < 0.05), high grade *P* < 0.05), Ki-67 index *P* < 0.05), HER2 positivity *P* < 0.05), HR negativity (*P* < 0.05)	No association	Pure DCIS only (n = 117)	Campbell et al. 2017 [113]
	No association	High TIL density was significantly associated with high nuclear grade (*P* < 0.001), ER negativity (*P* < 0.001), PR negativity (*P* < 0.001), HER2 positivity (*P* = 0.002), triple negativity (*P* = 0.001) and PD-L1 expression in both DCIS (*P* = 0.008) and surrounding TILs (*P* < 0.001)	High TIL density is significantly associated with recurrence (*P* = 0.012) and tended toward invasive recurrence (*P* = 0.086)	Pure DCIS (n = 186) & DCIS with microinvasion (n = 12)	Thike et al. 2020 [84]
**T cells (CD3+)**	Significantly higher proportion of T cells in mixed DCIS compared to pure DCIS (*P* = 0.0002)	High T cell infiltrate was significantly associated with high nuclear grade (*P* < 0.0001) and HER2+ DCIS (*P* = 0.0002)	No outcome data	Pure DCIS (n = 40) & mixed DCIS (n = 30)	Gil Del Alzazar et al. 2017 [85]
	Significantly higher proportion of CD3+ cells (*P* < 0.001) in mixed DCIS lesions compared to pure DCIS lesions	Not assessed	No outcome data	Pure DCIS (n = 19) & mixed DCIS (n = 11)	Mitchell et al. 2020 [92]
	Significantly higher proportion of CD3+ cells in mixed DCIS lesions compared to pure DCIS lesions (*P* = 0.001)	High stromal CD3+ expression is associated with high nuclear grade (*P* < 0.0001), comedonecrosis (*P* = 0.005), ER negativity (*P* < 0.0001), PR negativity (*P* < 0.0001), HER2 positivity (*P* < 0.0001) and high HIF1alpha expression (*P* < 0.0001)	No association	Pure DCIS (n = 508) & mixed DCIS (n = 192)	Toss et al. 2020 [114]
**Helper T cells (CD4+)**	Mixed DCIS (*P* = 0.004) and DCIS with microinvasion (*P* < 0.001) contained significantly higher proportion of CD4+ than pure DCIS	High CD4+ infiltrate was associated with high nuclear grade (*P* < 0.001), comedonecrosis (*P* = 0.004), ER negativity (*P* = 0.026), PR negativity (*P* = 0.027) and high Ki-67 index (*P* = 0.001) in pure DCIS. Infiltration of CD4+ (*P* = 0.001) TILs was higher in pure DCIS tumors with p53 expression	No association	Pure DCIS (n = 231) & mixed DCIS (n = 81)	Kim et al. 2020 [104]
	No difference between cohorts	Not assessed	No outcome data	Pure DCIS (n = 19) & mixed DCIS (n = 11)	Mitchell et al. 2020 [92]
	No difference in stromal or intratumoural CD4+	High stromal CD4+ cells associated with high nuclear grade (*P* < 0.0001), ER negativity (*P* < 0.0001), PR negativity (*P* < 0.0001), HER2 positivity (*P* < 0.0001) and high HIF1alpha expression (*P* < 0.0001). Intratumor CD4+ Not associated with HR status. Low Intratumoral CD4+ associated with increased tumor size (*P *= 0.016) and comedonecrosis (*P* = 0.032)	No association	Pure DCIS (n = 508) & mixed DCIS (n = 192)	Toss et al. 2020 [114]
	Not assessed	High grade DCIS had significantly higher percentages of CD4+ T cells (*P* < 0.01) compared to non-high grade DCIS. CD4+ cells were associated with VNPI, increased tumor size, high grade, Ki-67 index, HER2 positivity, HR negativity (*P* < 0.05)	No association	Pure DCIS only (n = 117)	Campbell et al. 2017 [113]
	CD4+ infiltrate was significantly higher in microinvasive lesions compared to pure DCIS lesions (*P* = 0.037)	High CD4+ infiltrate was significantly associated with high nuclear grade (*P* = 0.001), microinvasion (*P* = 0.037), ER negativity (*P* < 0.001), PR negativity (*P* = 0.001), HER2 positivity (*P* = 0.004), triple negativity (*P* = 0.023) and TIL PD‐L1 expression (*P* < 0.001)	Non-significant trend between high CD4+ T cell density and recurrence (*P* = 0.058) and significant association between high CD4+ infiltrate and shorter DFS (*P* = 0.001) and shorter DFS for invasive recurrence (*P* = 0.006)	Pure DCIS (n = 186) & DCIS with microinvasion (n = 12)	Thike et al. 2020 [84]
**Cytotoxic T cells (CD8+)**	In HR- tumors, DCIS-mixed contained significantly higher CD8+ T cell infiltrate than in pure DCIS (*P* = 0.009) and DCIS with microinvasion (*P* = 0.027)	High CD8+ infiltrate was associated with high nuclear grade (*P* = 0.001), comedonecrosis (*P* = 0.002), ER negativity (*P* = 0.025), PR negativity (*P* < 0.001) and high Ki-67 index (*P* = 0.009) in pure DCIS. Infiltration of CD8+ (*P* = 0.041) TILs was higher in PURE DCIS tumors with p53 expression. High infiltrate of CD8+ (*P* = 0.01) TILs was associated with HER2 positivity in pure DCIS tumors	No association	Pure DCIS (n = 231) & mixed DCIS (n = 81)	Kim et al. 2020 [104]
	There was a significantly higher proportion of CD8+ T cells (*P* < 0.0001) and CD8+ PD-1+ cells (*P* < 0.0001) in the immune infiltrate of mixed DCIS tissue than in pure DCIS tissue	Not assessed	No outcome data	Pure DCIS (n = 19) & mixed DCIS (n = 11)	Mitchell et al. 2020 [92]
	Mixed DCIS lesions contained significantly higher stromal CD8+ (*P* < 0.0001) and PD-1 (*P* < 0.0001). Intratumorally, mixed DCIS lesions contained significantly higher PD-1 + (*P* = 0.025) compared to pure DCIS lesions. No difference in CD8+ Intratumorally	High stromal CD8+ cells associated with younger age (*P* = 0.01), high nuclear grade (*P* < 0.0001), Comedonecrosis (*P* = 0.037), ER negativity (*P* < 0.0001), PR negativity (*P* < 0.0001), HER2 positivity (*P* = 0.001) and high Hif1alpha expression (*P* < 0.0001). High stromal PD1 + expression was associated with high nuclear grade (*P* < 0.0001), comedonecrosis (*P* = 0.001), ER negativity (*P* < 0.0001), PR negativity (*P* < 0.0001), HER2 positivity (*P* < 0.0001), and high HIf1alpha expression (*P* < 0.0001). High Intratumoral PD-1 + expression was associated with higher nuclear grade, HER2 positivity, and higher expression of HIF-1α	No association	Pure DCIS (n = 508) & mixed DCIS (n = 192)	Toss et al. 2020 [114]
	Not assessed	CD8+ infiltrate was associated with lymph node involvement (*P* = 1.4 × 10^–7^) and high CD8+ /FOXP3+ ratio (*P* = 0.036)	Low CD8+ Infiltrate was associated with relapse (*P* = 0.036)	Pure DCIS (n = 199) & mixed DCIS (49)	Semeraro et al. 2016 [115]
	Not assessed	CD8+ cells were associated with high grade, HER2 positivity and HR negativity (*P* < 0.05)	Highest risk of recurrence was cases with low numbers of activated CD8+ HLADR+ cells (P < 0.00001). Cases with high numbers of CD8+ HLADR+ and low numbers of CD8+ HLADR- cells were at low risk of recurrence	Pure DCIS only (n = 117)	Campbell et al. 2017 [113]
**Regulatory T cells (CD4+ and FOXP3+)**	DCIS-microinvasion contained significantly higher FOXP3+ infiltrate than pure DCIS (*P* < 0.001). DCIS-mixed contained significantly higher FOXP3+ (*P* = 0.005) TIL infiltrate than in pure DCIS. No Difference found between DCIS-microinvasion and DCIS-mixed	High FOXP3+ infiltrate was associated with high nuclear grade (*P* < 0.001), comedonecrosis (*P* = 0.008), ER negativity (*P* = 0.004), PR negativity (*P* = 0.002) and high Ki-67 index (*P* < 0.001) in pure DCIS. Infiltration of FOXP3+ (*P* = 0.016) Tils was higher in PURE DCIS tumors with p53 expression. High infiltrate of FOXP3+ (*P* = 0.004) TILs was associated with HER2 positivity in pure DCIS tumors	For pure DCIS patients, high FOXP3+ infiltrate associated with decreased recurrence-free survival (*P* = 0.002)	Pure DCIS (n = 231) & mixed DCIS (n = 81)	Kim et al. 2020 [104]
	Not assessed	High numbers of FOXP3+ cells are associated with high nuclear grade (*P* < 0.001), lymph node involvement (*P* = 0.01), ER negativity (*P* = 0.001)	DCIS tumors with high number of Tregs indicated a worse recurrence free survival (*P* = 0.04)	Pure DCIS only (n = 62)	Bates et al. 2006 [121]
	Mixed DCIS lesions contained significantly higher stromal FOXP3 + (P = 0.016)	High stromal FOXP3+ cells were associated with high nuclear grade (*P* < 0.0001), ER negativity (*P* < 0.0001), PR negativity (*P* < 0.0001), HER2 positivity (*P* < 0.0001) and high HIF1alpha expression (*P* < 0.0001). High Intratumoural expression of FOXP3+ was associated with high nuclear grade (*P* = 0.02), ER negativity (*P* = 0.049), PR negativity (*P* = 0.009), HER2 positivity (*P* = 0.002) and high HIF1alpha expression (*P* < 0.0001)	High expression of stromal FOXP3+ T cells (*P *= 0.025) was associated with all recurrences	Pure DCIS (n = 508) & mixed DCIS (n = 192)	Toss et al. 2020 [114]
	Not assessed	No difference in proportion of FOXP3+ T cells between DCIS grades	No outcome data	Mixed DCIS only (n = 32)	Lal et al. 2013 [102]
	There was a significantly lower proportion of FOXP3+ T cells in the immune infiltrate of mixed DCIS tissue than in pure DCIS tissue (*P* < 0.0001)	Not assessed	No outcome data	Pure DCIS (n = 19) & mixed DCIS (n = 11)	Mitchell et al. 2020 [92]
	Not assessed	High grade DCIS had significantly higher percentages of FOXP3+ T cells (*P* < 0.01) than non-high-grade DCIS. FOXP3 infiltrates were significantly and positively associated with VNPI, comedonecrosis, Ki-67 index, HER2 positivity and HR negativity	No association	Pure DCIS only (n = 117)	Campbell et al. 2017 [113]
**T cell ratio**	Not assessed	There were more CD4+ T cells than CD8+ T cells in the HR+ pure DCIS (*P* < 0.001)	High FOXP3+ /CD8+ TIL ratio (*P* = 0.023) and high FOXP3+ /CD4+ T TIL (*P* = 0.036) ratio associated with decreased recurrence free survival. There were more CD4 + T cells than CD8+ T cells in the HR+ pure DCIS (*P* < 0.001)	Pure DCIS (n = 231) & mixed DCIS (n = 81)	Kim et al. 2020 [104]
	No association	High CD4/CD8 ratios were significantly associated with PD-L1 expression in both DCIS (*P* = 0.035) and TILs (*P* < 0.001) and trended toward high nuclear grade (*P* = 0.052)	High CD4/CD8+ T cell ratio was associated with shorter disease-free survival (*P* = 0.028) and shorter disease-free survival for invasive recurrence (*P* = 0.02)	Pure DCIS (n = 186) & DCIS with microinvasion (n = 12)	Thike et al. 2020 [84]
	Not assessed	High CD8/FOXP3+ ratio associated with lymph node involvement and high CD8+ infiltrate	Low CD8/FOXP3 ratio associated with relapse (*P* = 0.00377)	Pure DCIS (n = 199) & mixed DCIS (49)	Semeraro et al. 2016 [115]
**PD-L1+ cells**	No difference in PD-L1+ infiltrate between pure, DCIS with microinvasion and DCIS mixed	High PD-L1+ infiltrate was associated with high nuclear grade (*P* < 0.001), comedonecrosis (*P* < 0.001), ER negativity (*P* = 0.043), PR negativity (*P* = 0.019) and high Ki-67 index (*P* < 0.001) in pure DCIS	For pure DCIS patients, high PD-L1+ immune cells found to be associated with decreased recurrence free survival (*P* = 0.018)	Pure DCIS (n = 231) & mixed DCIS (n = 81)	Kim et al. 2020 [104]
	No association	100% of Triple negative DCIS had high expression of PD-L1 + on tumor infiltrating lymphocytes (*P* = 0.0008). 80% of DCIS containing PD-L1 − tumor infiltrating lymphocytes occurred in older patients (*P* = 0.02). DCIS with low tumor infiltrating lymphocyte density scores were more likely to be PD-L1 − than PD -L1 + (*P* = 0.004)	All DCIS patients with concurrent IDC or recurrent DCIS had high PDL-1+ TILs, but not statistically significant	Pure DCIS (n = 24) & mixed DCIS (n = 3)	Thompson et al. 2016 [105]
	Mixed DCIS lesions contained significantly higher stromal PD-L1 expression (*P* = 0.019)	High stromal PD-L1+ expression was associated with high nuclear grade (*P* < 0.0001), ER negativity(*P* < 0.0001), PR negativity (*P* < 0.0001), HER2 positivity (*P* < 0.0001), and High HIF1alpha expression (P < 0.0001)	High expression of stromal PD-L1+ was associated with shorter LRFI for all recurrences (*P* < 0.0001) and invasive recurrences (*P* = 0.032)	Pure DCIS (n = 508) & mixed DCIS (n = 192)	Toss et al. 2020 [114]
	Not assessed	PD-L1+ expressing tumors (in any compartment) were more likely to be ER- (*P* = 0.019) and HER2 positive (*P* = 0.046). Tumor cell PD-L1 positivity were more likely to be high grade (*P* = 0.033) (but no association between grade and immune PD-L1 expression). Comedonecrosis was not associated with PD-L1 expression	PDL1 expression was not related to recurrence (79 cases)	Pure DCIS only (n = 79)	Hendry et al. 2017 [127]
	Not assessed	PD-L1 + expression is significantly higher in HER2+ tumors compared to HER2- tumors (*P* = 0.02)	No outcome data	Pure DCIS only (n = 85)	Ubago et al. 2019 [118]
**Macrophages** **(CD68+ , CD163+ or CD115+)**	Not assessed	High grade DCIS had significantly higher percentages of CD68+ macrophages (*P* < 0.01), CD68+ PCNA+ macrophages (P < 0.05) than non-high-grade DCIS. CD68+ Macrophages were correlated with high VPNI, palpability, high grade, comedonecrosis, Ki-67 positivity, HR negativity (all P < 0.05). CD68+ MRC1+ cells were not significantly correlated with any clinical parameters (M2 type macrophage). CD115 + macrophages were associated with high Ki-67 and HER2 positivity (P < 0.05)	Cases with high CD8+ HLADR+ cells, but also high numbers of non-activated CD8+ HLADR− cells and high numbers of CD115+ cells were also at a high risk for recurrence (*P* < 0.0001) and cases with high CD8+ HLADR+ cells, high CD8+ HLADR− cells, and low CD115+ cells were at a low risk for recurrence	Pure DCIS only (n = 117)	Campbell et al. 2017 [113]
	Not assessed	High CD68+ macrophage density was associated with high nuclear grade (*P* < 0.001), estrogen receptor (ER) negativity (*P* = 0.029), progesterone receptor (PR) negativity (*P* = 0.008) and human epidermal growth factor receptor 2 (HER2) positivity (*P* < 0.001). High CD163+ macrophage density was associated with high nuclear grade (*P* = 0.003), microinvasion (*P* = 0.01), ER negativity (*P* < 0.001), PR negativity (*P* = 0.001), HER2 positivity (*P* = 0.001) and triple negativity (*P* = 0.022)	High CD68+ macrophage density was significantly associated with worse DFS for ipsilateral invasive recurrence (*P* = 0.004). High CD163+ macrophage density showed significantly worse DFS for both DCIS recurrence (*P* = 0.001) and ipsilateral invasive recurrence (*P* = 0.001)	Pure DCIS (n = 186) & DCIS with microinvasion (n = 12)	Chen et al. 2020 [117]
	No association	There was no correlation between Intratumoral CD163 expression and DCIS grade but there was a trend for higher stromal CD163 expression in high grade DCIS (although not statistically significant)	No outcome data	Pure DCIS (n = 30) & mixed DCIS (n = 27)	Hoskoppal et al. 2018 [128]
**B cells (CD20+)**	Mixed DCIS lesions contained significantly higher stromal CD20 (*P* < 0.0001) but contained significantly less intratumorally CD20 (*P* = 0.007) compared to pure DCIS	High stromal expression of CD20+ was associated with HR negativity but high intratumoural CD20+ was associated with ER and PR positivity. High stromal CD20+ was associated with high nuclear grade (*P* < 0.0001), comedonecrosis (*P* = 0.002), ER negativity (*P* < 0.0001), PR negativity (*P* < 0.0001), HER2 positivity (*P* = 0.001) and high HIF1alpha expression (*P* < 0.0001). High intratumoural CD20+ expression was associated with ER positivity (*P* = 0.02), PR positivity (*P* = 0.01) and low HIF-1alpha (*P* = 0.014)	No association	Pure DCIS (n = 508) & mixed DCIS (n = 192)	Toss et al. 2020 [114]
	Not assessed	High grade DCIS had significantly higher percentages of CD20+ B cells (*P* < 0.01) compared to non-high grade DCIS. CD20+ cells were associated with VNPI, increased tumor size, high grade, Ki-67 index, HER2 positivity, HR negativity (*P* < 0.05)	No association	Pure DCIS only (n = 117)	Campbell et al. 2017 [113]
	Significantly more CD20+ B cells intratumorally in pure DCIS compared to mixed DCIS (*P* = 0.04)	In pure DCIS cases, Dense CD20+ B cell infiltrate was associated significantly with larger tumor size (*P *= 0.016), hormone receptor (ER/PR) ‐negative tumors (*P* = 0.008) and HER2‐positive status (*P* = 0.01). In the mixed cohort, higher B cell infiltrate was associated significantly with larger (invasive and in situ) tumor size (*P* = 0.019), higher invasive tumor grade (*P* = 0.005), the presence of DCIS necrosis (*P* = 0.042), lymphovascular invasion (*P* = 0.022), lymph node metastases (*P* = 0.033), negative ER/PR status (*P* = 0.04) and positive HER2 status (*P* = 0.008). A higher number of plasma cells was associated significantly with ER/PR‐negative tumors (*P* = 0.01) and HER2 positivity (*P* = 0.019). No association between intratumoural B cells infiltrate and clinical features	DCIS lesions with less stromal B lymphocytes were significantly associated with a longer RFS (*P* = 0.01). Intratumoral TIL‐Bs did not show a significant association with patient outcome	Pure DCIS (n = 36) & mixed DCIS (n = 44)	Miligy et al. 2017 [116]

*DCIS* Ductal Carcinoma in situ, *RFI* Recurrence-free interval, *DFS* Disease free survival, *TILs* Tumor infiltrating lymphocytes, *PD-L1* Programmed cell death ligand 1

## Conclusion

Although non-invasive, DCIS may progress to invasive disease if left untreated. Since there is no accurate means of predicting which patients’ DCIS will progress to invasive disease, every patient is presently considered to have the same risk of progression. Clinically, this means patients are treated with surgery, radiotherapy and in some cases endocrine therapy. The current management of DCIS patients undoubtedly mitigates the risk of recurrence and progression. However, considering that up to 60% of untreated cases will never progress to IDC and will instead remain indolent*,* there is substantial overtreatment of DCIS patients. The co-morbidities, toxicities, mental and financial impacts from the widespread overtreatment of DCIS patients motivates the identification of prognostic biomarkers that can distinguish between low and high-risk patients. Patients identified as having a high risk of invasive progression would benefit from extra medical intervention, including radiotherapy and systemic treatment, whereas those identified as having a low risk may require minimal treatment such as surgery alone or active surveillance. To identify predictive markers, the transition from DCIS to invasive ductal carcinoma needs to be better understood. There is increasing evidence for the critical role the microenvironment plays in tumor progression across all cancers. Such information can assist in classifying tumors and predicting treatment response. For DCIS, the limited mutational difference between in situ and invasive tumor cells, suggests it is likely that other cell types govern the transition. While the tumor cells themselves largely remain the same, stromal and immune cells are phenotypically different in DCIS and IDC tissue. Furthermore, some of the phenotypic abnormalities in these cells are evident to a greater extent in DCIS tissue that has invasive counterparts. Perhaps early expression changes can predict disease outcome. Future investigation involving larger cohorts combined with long-term follow up may generate noteworthy observations that could assist in creating a prognostic tool to accurately predict the fate of DCIS tumor cells. Such a tool would be implemented at the time of DCIS diagnosis and would optimize individual patient therapy, reducing the incidence of overtreatment. It is clear that the clinical value of the information embodied in the DCIS microenvironment has been under-exploited to-date and that implementation of a whole-tissue strategy to guide treatment has the potential to substantially improve personalized management of DCIS.

## References

[1] Moumen M, Chiche A, Cagnet S, Petit V (2011). The mammary myoepithelial cell. Int J Dev Biol.

[2] Mariotti C (2018). Ductal Carcinoma in Situ of the Breast.

[3] Siegel RL, Miller KD, Jemal A (2020). Cancer statistics, 2020. CA Cancer J Clin.

[4] Australian Institute of Health and Welfare (2021). BreastScreen Australia monitoring report 2021.

[5] Ryser MD, Weaver DL, Zhao F, Worni M (2019). Cancer outcomes in DCIS patients without locoregional treatment. J Natl Cancer Inst.

[6] Worni M, Akushevich I, Greenup R, Sarma D (2015). Trends in treatment patterns and outcomes for ductal carcinoma in situ. J Natl Cancer Inst.

[7] Group EBCTC (2010). Overview of the randomized trials of radiotherapy in ductal carcinoma in situ of the breast. JNCI Monogr.

[8] Solin LJ, Gray R, Hughes LL, Wood WC (2015). Surgical excision without radiation for ductal carcinoma in situ of the breast: 12-year results from the ECOG-ACRIN E5194 study. J Clin Oncol.

[9] Raldow AC, Sher D, Chen AB, Recht A (2016). Cost Effectiveness of the oncotype DX DCIS score for guiding treatment of patients with ductal carcinoma in situ. J Clin Oncol.

[10] Polyak K (2010). Molecular markers for the diagnosis and management of ductal carcinoma in situ. J Natl Cancer Inst Monogr.

[11] Gorringe KL, Fox SB (2017). Ductal carcinoma in situ biology, biomarkers, and diagnosis. Front Oncol.

[12] O'Grady S, Morgan MP (2018). Microcalcifications in breast cancer: From pathophysiology to diagnosis and prognosis. BBA - Reviews on Cancer.

[13] Giannakeas V, Sopik V, Narod SA (2020). Association of a diagnosis of ductal carcinoma in situ with death from breast cancer. JAMA Netw Open.

[14] Castellano I, Metović J, Bussone R, Grilz G, Mariotti C (2018). DCIS: Pathology and biological features. Ductal Carcinoma in Situ of the Breast.

[15] The Consensus Conference Committee. Consensus conference on the classification of ductal carcinoma in Situ. Cancer. 1997;80(9):1798–802. 10.1002/(SICI)1097-0142(19971101)80:9<1798::AID-CNCR15>3.0.CO;2-0. 10.1002/(sici)1097-0142(19971101)80:9<1798::aid-cncr15>3.0.co;2-0 9351550

[16] Champion CD, Ren Y, Thomas SM, Fayanju OM (2019). DCIS with microinvasion: Is it in situ or invasive disease?.. Ann Surg Oncol.

[17] Sanders ME, Schuyler PA, Simpson JF, Page DL (2015). Continued observation of the natural history of low-grade ductal carcinoma in situ reaffirms proclivity for local recurrence even after more than 30 years of follow-up. Mod Pathol.

[18] Collins LC, Tamimi RM, Baer HJ, Connolly JL (2005). Outcome of patients with ductal carcinoma in situ untreated after diagnostic biopsy: results from the Nurses' Health Study. Cancer.

[19] Maxwell AJ, Clements K, Hilton B, Dodwell DJ (2018). Risk factors for the development of invasive cancer in unresected ductal carcinoma in situ. Eur J Surg Oncol.

[20] Erbas B, Provenzano E, Armes J, Gertig D (2006). The natural history of ductal carcinoma in situ of the breast: a review. Breast Cancer Res Treat.

[21] Cuzick J, Sestak I, Pinder SE, Ellis IO (2011). Effect of tamoxifen and radiotherapy in women with locally excised ductal carcinoma in situ: long-term results from the UK/ANZ DCIS trial. Lancet Oncol.

[22] Donker M, Litiere S, Werutsky G, Julien JP (2013). Breast-conserving treatment with or without radiotherapy in ductal carcinoma In Situ: 15-year recurrence rates and outcome after a recurrence, from the EORTC 10853 randomized phase III trial. J Clin Oncol.

[23] Wapnir IL, Dignam JJ, Fisher B, Mamounas EP (2011). Long-term outcomes of invasive ipsilateral breast tumor recurrences after lumpectomy in NSABP B-17 and B-24 randomized clinical trials for DCIS. J Natl Cancer Inst.

[24] Wärnberg F, Garmo H, Emdin S, Hedberg V (2014). Effect of radiotherapy after breast-conserving surgery for ductal carcinoma in situ: 20 years follow-up in the randomized SweDCIS Trial. Journal of clinical oncology : official journal of the American Society of Clinical Oncology.

[25] Elshof LE, Schaapveld M, Schmidt MK, Rutgers EJ (2016). Subsequent risk of ipsilateral and contralateral invasive breast cancer after treatment for ductal carcinoma in situ: incidence and the effect of radiotherapy in a population-based cohort of 10,090 women. Breast Cancer Res Treat.

[26] McCormick B, Winter K, Hudis C, Kuerer HM (2015). RTOG 9804: a prospective randomized trial for good-risk ductal carcinoma in situ comparing radiotherapy with observation. J Clin Oncol.

[27] Narod SA, Iqbal J, Giannakeas V, Sopik V (2015). Breast cancer mortality after a diagnosis of ductal carcinoma in situ. JAMA Oncol.

[28] Rakovitch E, Nofech-Mozes S, Hanna W, Sutradhar R (2018). Omitting radiation therapy after lumpectomy for pure DCIS does not reduce the risk of salvage mastectomy. The Breast.

[29] Thompson AM, Clements K, Cheung S, Pinder SE (2018). Management and 5-year outcomes in 9938 women with screen-detected ductal carcinoma in situ: the UK Sloane Project. Eur J Cancer.

[30] Trotti A, Byhardt R, Stetz J, Gwede C (2000). Common toxicity criteria: version 2.0. an improved reference for grading the acute effects of cancer treatment: impact on radiotherapy. Int J Radiat Oncol Biol Phys.

[31] Coates AS, Winer EP, Goldhirsch A, Gelber RD (2015). Tailoring therapies—improving the management of early breast cancer: St Gallen International Expert Consensus on the Primary Therapy of Early Breast Cancer 2015. Ann Oncol.

[32] Lari SA, Kuerer HM (2011). Biological markers in DCIS and risk of breast recurrence: A systematic review. J Cancer.

[33] Kim SY, Jung SH, Kim MS, Baek IP (2015). Genomic differences between pure ductal carcinoma in situ and synchronous ductal carcinoma in situ with invasive breast cancer. Oncotarget.

[34] Francis A, Thomas J, Fallowfield L, Wallis M (2015). Addressing overtreatment of screen detected DCIS; the LORIS trial. Eur J Cancer.

[35] Elshof LE, Tryfonidis K, Slaets L, van Leeuwen-Stok AE (2015). Feasibility of a prospective, randomised, open-label, international multicentre, phase III, non-inferiority trial to assess the safety of active surveillance for low risk ductal carcinoma in situ – The LORD study. Eur J Cancer.

[36] Hwang ES, Hyslop T, Lynch T, et al. The COMET (Comparison of Operative versus Monitoring and Endocrine Therapy) trial: a phase III randomised controlled clinical trial for low-risk ductal carcinoma in situ (DCIS). BMJ Open. 2019;9:e026797. 10.1136/bmjopen-2018-026797. 10.1136/bmjopen-2018-026797 PMC6429899 30862637

[37] NIPH: NIPH Clinical Trials Search. https://rctportal.niph.go.jp/en/detail?trial_id=UMIN000028298. 2017. Accessed 11 May 2020.

[38] Lippey J, Spillane A, Saunders C (2016). Not all ductal carcinoma in situ is created equal: can we avoid surgery for low-risk ductal carcinoma in situ?. ANZ J Surg.

[39] Takada K, Kashiwagi S, Asano Y, Goto W (2020). Factors predictive of invasive ductal carcinoma in cases preoperatively diagnosed as ductal carcinoma in situ. BMC Cancer.

[40] Grimm L, Ryser M, Partridge A, Thompson A (2017). Surgical upstaging rates for vacuum assisted biopsy proven DCIS: Implications for active surveillance trials. Ann Surg Oncol.

[41] Pilewskie M, Stempel M, Rosenfeld H, Eaton A (2016). Do LORIS trial eligibility criteria identify a ductal carcinoma in situ patient population at low risk of upgrade to invasive carcinoma?.. Ann Surg Oncol.

[42] Podoll MB, Reisenbichler ES, Roland L, Bruner A (2018). Feasibility of the less is more approach in treating low-risk ductal carcinoma in situ diagnosed on core needle biopsy: Ten-year review of ductal carcinoma in situ upgraded to invasion at surgery. Arch Pathol Lab Med.

[43] Lo PK, Zhang Y, Yao Y, Wolfson B (2017). Tumor-associated myoepithelial cells promote the invasive progression of ductal carcinoma in situ through activation of TGFbeta signaling. J Biol Chem.

[44] Weigelt B, Bissell MJ (2008). Unraveling the microenvironmental influences on the normal mammary gland and breast cancer. Semin Cancer Biol.

[45] Van Seijen M, Lips EH, Thompson AM, Nik-Zainal S (2019). Ductal carcinoma in situ: to treat or not to treat, that is the question. Br J Cancer.

[46] Casasent AK, Edgerton M, Navin NE (2017). Genome evolution in ductal carcinoma in situ: Invasion of the clones. J Pathol.

[47] Ma X-J, Salunga R, Tuggle JT, Gaudet J (2003). Gene expression profiles of human breast cancer progression. PNAS.

[48] Casasent AK, Schalck A, Gao R, Sei E (2018). Multiclonal invasion in breast tumors identified by topographic single cell sequencing. Cell.

[49] Ang DC, Warrick AL, Shilling A, Beadling C (2014). Frequent phosphatidylinositol-3-kinase mutations in proliferative breast lesions. Mod Pathol.

[50] Mardekian SKMD, Bombonati AMD, Palazzo JPMD (2015). Ductal carcinoma in situ of the breast: The importance of morphologic and molecular interactions. Hum Pathol.

[51] Gupta SK, Douglas-Jones AG, Fenn N, Morgan JM (1997). The clinical behavior of breast carcinoma is probably determined at the preinvasive stage (Ductal carcinoma in Situ). Cancer.

[52] Afghahi A, Forgó E, Mitani AA, Desai M (2015). Chromosomal copy number alterations for associations of ductal carcinoma in situ with invasive breast cancer. Breast Cancer Res.

[53] Gorringe KL, Hunter SM, Pang J-M, Opeskin K (2015). Copy number analysis of ductal carcinoma in situ with and without recurrence. Mod Pathol.

[54] Lin C-Y, Vennam S, Purington N, Lin E (2019). Genomic landscape of ductal carcinoma in situ and association with progression. Breast Cancer Res Treat.

[55] Brock EJ, Ji K, Shah S, Mattingly RR (2019). In vitro models for studying invasive transitions of ductal carcinoma in situ. J Mammary Gland Biol Neoplasia.

[56] Behbod F, Kittrell FS, LaMarca H, Edwards D (2009). An intraductal human-in-mouse transplantation model mimics the subtypes of ductal carcinoma in situ. Breast Cancer Res.

[57] Sflomos G, Dormoy V, Metsalu T, Jeitziner R (2016). A preclinical model for ERα-positive breast cancer points to the epithelial microenvironment as determinant of luminal phenotype and hormone response. Cancer Cell.

[58] Behbod F, Gomes AM, Machado HL (2018). Modeling human ductal carcinoma in situ in the mouse. J Mammary Gland Biol Neoplasia.

[59] Arendt LM, Rugowski DE, Grafwallner-Huseth TA, Garcia-Barchino MJ (2011). Prolactin-induced mouse mammary carcinomas model estrogen resistant luminal breast cancer. Breast Cancer Res.

[60] Lin EY, Jones JG, Li P, Zhu L (2003). Progression to malignancy in the polyoma middle T oncoprotein mouse breast cancer model provides a reliable model for human diseases. Am J Pathol.

[61] Chan SR, Vermi W, Luo J, Lucini L, et al. STAT1-deficient mice spontaneously develop estrogen receptor α-positive luminal mammary carcinomas. Breast Cancer Res BCR. 2012;14(1):R16-R. 10.1186/bcr3100. 10.1186/bcr3100 PMC3496133 22264274

[62] Verbeke S, Richard E, Monceau E, Schmidt X (2014). Humanization of the mouse mammary gland by replacement of the luminal layer with genetically-engineered preneoplastic human cells. Breast Cancer Res.

[63] Chan SR, Rickert CG, Vermi W, Sheehan KC (2014). Dysregulated STAT1-SOCS1 control of JAK2 promotes mammary luminal progenitor cell survival and drives ERα(+) tumorigenesis. Cell Death Differ.

[64] Sameni M, Cavallo-Medved D, Franco OE, Chalasani A (2017). Pathomimetic avatars reveal divergent roles of microenvironment in invasive transition of ductal carcinoma in situ. Breast Cancer Res BCR.

[65] Brummer G, Acevedo DS, Hu Q, Portsche M (2018). Chemokine signaling facilitates early-stage breast cancer survival and invasion through fibroblast-dependent mechanisms. Molecular cancer research : MCR.

[66] Shamliyan T, Wang S-Y, Virnig BA, Tuttle TM (2010). Association between patient and tumor characteristics with clinical outcomes in women with ductal carcinoma in situ. JNCI Monographs.

[67] Wang S-Y, Shamliyan T, Virnig B, Kane R (2011). Tumor characteristics as predictors of local recurrence after treatment of ductal carcinoma in situ: a meta-analysis. Breast Cancer Res Treat.

[68] Zhang X, Dai H, Liu B, Song F (2016). Predictors for local invasive recurrence of ductal carcinoma in situ of the breast: a meta-analysis. Eur J Cancer Prev.

[69] Visser LL, Groen EJ, van Leeuwen FE, Lips EH (2019). predictors of an invasive breast cancer recurrence after DCIS: a systematic review and meta-analyses. Cancer Epidemiol Biomark Prev.

[70] Silverstein MJ, Lagios MD (2010). Choosing treatment for patients with ductal carcinoma in situ: fine tuning the University of Southern California/Van Nuys Prognostic Index. J Natl Cancer Inst Monogr.

[71] Cobain EF, Hayes DF (2015). Indications for prognostic gene expression profiling in early breast cancer. Curr Treat Options Oncol.

[72] Solin LJ, Gray R, Baehner FL, Butler SM (2013). A multigene expression assay to predict local recurrence risk for ductal carcinoma in situ of the breast. J Natl Cancer Inst.

[73] Bremer T, Whitworth PW, Patel R, Savala J (2018). A biological signature for breast ductal carcinoma in situ to predict radiotherapy benefit and assess recurrence risk. Clin Cancer Res.

[74] Weinmann S, Leo M, Francisco M, Jenkins C, et al. Validation of a ductal carcinoma in situ biomarker profile for risk of recurrence after breast-conserving surgery with and without radiation therapy. Clin Cancer Res. 2020;clincanres.1152.2019. 10.1158/1078-0432.CCR-19-1152. 10.1158/1078-0432.CCR-19-1152 32341032

[75] Wadsten C, Whitworth PW, Patel R, Savala J (2019). Risk stratification in earl- stage luminal breast cancer patients treated with and without RT. J Clin Oncol.

[76] Wärnberg F, Garmo H, Folkvaljon Y, Holmberg L, et al. Abstract GS5–08: A validation of DCIS biological risk profile in a randomised study for radiation therapy with 20 year follow-up (SweDCIS). Cancer Res. 2018;78(4 Supplement):GS5–08-GS5-. 10.1158/1538-7445.Sabcs17-gs5-08.

[77] Rakovitch E, Nofech-Mozes S, Hanna W, Baehner FL (2015). A population-based validation study of the DCIS Score predicting recurrence risk in individuals treated by breast-conserving surgery alone. Breast Cancer Res Treat.

[78] Hanahan D, Weinberg RA (2011). Hallmarks of cancer: the next generation. Cell.

[79] Logullo ÂF, Nonogaki S, Pasini FS, De Toledo Osório CAB (2010). Concomitant expression of epithelial-mesenchymal transition biomarkers in breast ductal carcinoma: Association with progression. Oncol Rep.

[80] Nallanthighal S, Heiserman JP, Cheon DJ. The role of the extracellular matrix in cancer stemness. Front Cell Dev Biol. 2019;7:86. 10.3389/fcell.2019.00086. 10.3389/fcell.2019.00086 PMC6624409 31334229

[81] Miligy IM, Gorringe KL, Toss MS, Al-Kawaz AA (2018). Thioredoxin-interacting protein is an independent risk stratifier for breast ductal carcinoma in situ. Mod Pathol.

[82] Toss MS, Miligy IM, Gorringe KL, McCaffrey L (2019). Legumain is an independent predictor for invasive recurrence in breast ductal carcinoma in situ. Mod Pathol.

[83] Kim A, Lee SJ, Kim YK, Park WY (2017). Programmed death-ligand 1 (PD-L1) expression in tumour cell and tumour infiltrating lymphocytes of HER2-positive breast cancer and its prognostic value. Sci Rep.

[84] Thike AA, Chen X, Koh VCY, Binte Md Nasir ND (2020). Higher densities of tumour-infiltrating lymphocytes and CD4+ T cells predict recurrence and progression of ductal carcinoma in situ of the breast. Histopathology.

[85] Gil Del Alcazar CR, Huh SJ, Ekram MB, Trinh A (2017). Immune escape in breast cancer during to invasive carcinoma transition. Cancer Discov.

[86] Russell TD, Jindal S, Agunbiade S, Gao D (2015). Myoepithelial cell differentiation markers in ductal carcinoma in situ progression. Am J Pathol.

[87] Toussaint J, Durbecq V, Altintas S, Doriath V, et al. Low CD10 mRNA expression identifies high-risk ductal carcinoma in situ (DCIS). PLoS One. 2010;5(8):e12100. 10.1371/journal.pone.0012100. 10.1371/journal.pone.0012100 PMC2938371 20856894

[88] Rohilla M, Bal A, Singh G, Joshi K (2015). Phenotypic and functional characterization of ductal carcinoma in situ-associated myoepithelial cells. Clin Breast Cancer.

[89] Zhang RR, Man Y-G, Vang R, Saenger JS (2003). A subset of morphologically distinct mammary myoepithelial cells lacks corresponding immunophenotypic markers. Breast Cancer Res.

[90] Hilson JB, Schnitt SJ, Collins LC (2009). Phenotypic alterations in ductal carcinoma in situ-associated myoepithelial cells: biologic and diagnostic implications. Am J Surg Pathol.

[91] Werling RW, Hwang H, Yaziji H, Gown AM (2003). Immunohistochemical distinction of invasive from noninvasive breast lesions: a comparative study of p63 versus calponin and smooth muscle myosin heavy chain. Am J Surg Pathol.

[92] Mitchell E, Jindal S, Chan T, Narasimhan J (2020). Loss of myoepithelial calponin-1 characterizes high-risk ductal carcinoma in situ cases, which are further stratified by T cell composition. Mol Carcinog.

[93] Allen MD, Thomas GJ, Clark S, Dawoud MM (2014). Altered microenvironment promotes progression of preinvasive breast cancer: myoepithelial expression of αvβ6 integrin in DCIS identifies high-risk patients and predicts recurrence. Clin Cancer Res.

[94] Sarper M, Allen MD, Gomm J, Haywood L, et al. Loss of MMP-8 in ductal carcinoma in situ (DCIS)-associated myoepithelial cells contributes to tumour promotion through altered adhesive and proteolytic function. Breast Cancer Res. 2017;19(1):33. 10.1186/s13058-017-0822-9. 10.1186/s13058-017-0822-9 PMC5363009 28330493

[95] Duivenvoorden HM, Rautela J, Edgington-Mitchell LE, Spurling A (2017). Myoepithelial cell-specific expression of stefin A as a suppressor of early breast cancer invasion. J Pathol.

[96] Toss MS, Miligy IM, Haj-Ahmad R, Gorringe KL (2019). The prognostic significance of lysosomal protective protein (cathepsin A) in breast ductal carcinoma in situ. Histopathology.

[97] Toss M, Miligy I, Gorringe K, Mittal K (2020). Prognostic significance of cathepsin V (CTSV/CTSL2) in breast ductal carcinoma in situ. J Clin Pathol.

[98] Toss MS, Miligy IM, Gorringe KL, AlKawaz A (2018). Prolyl-4-hydroxylase Α subunit 2 (P4HA2) expression is a predictor of poor outcome in breast ductal carcinoma in situ (DCIS). Br J Cancer.

[99] Toss M, Miligy I, Gorringe K, Aleskandarany M (2019). Collagen (XI) alpha-1 chain is an independent prognostic factor in breast ductal carcinoma in situ. Mod Pathol.

[100] Allinen M, Beroukhim R, Cai L, Brennan C (2004). Molecular characterization of the tumor microenvironment in breast cancer. Cancer Cell.

[101] Salemme V, Centonze G, Cavallo F, Defilippi P, et al. The crosstalk between tumor cells and the immune microenvironment in breast cancer: Implications for immunotherapy. Front Oncol. 2021;11:610303. 10.3389/fonc.2021.610303. 10.3389/fonc.2021.610303 PMC7991834 33777750

[102] Lal A, Chan L, DeVries S, Chin K (2013). FOXP3-positive regulatory T lymphocytes and epithelial FOXP3 expression in synchronous normal, ductal carcinoma in situ, and invasive cancer of the breast. Breast Cancer Res Treat.

[103] Hussein MR, Hassan HI (2006). Analysis of the mononuclear inflammatory cell infiltrate in the normal breast, benign proliferative breast disease, in situ and infiltrating ductal breast carcinomas: preliminary observations. J Clin Pathol.

[104] Kim M, Chung YR, Kim HJ, Woo JW (2020). Immune microenvironment in ductal carcinoma in situ: a comparison with invasive carcinoma of the breast. Breast Cancer Res.

[105] Thompson E, Taube JM, Elwood H, Sharma R (2016). The immune microenvironment of breast ductal carcinoma in situ. Mod Pathol.

[106] Lv S, Wang S, Qiao G, Wang X (2019). Functional CD3+CD8+PD1− T cell accumulation and PD-L1 expression increases during tumor invasion in DCIS of the breast. Clin Breast Cancer.

[107] Shou D, Wen L, Song Z, Yin J, et al. Suppressive role of myeloid-derived suppressor cells (MDSCs) in the microenvironment of breast cancer and targeted immunotherapies. Oncotarget. 2016;7(39):64505–11. 10.18632/oncotarget.11352. 10.18632/oncotarget.11352 PMC5325458 27542274

[108] Cole K, Pravoverov K, Talmadge JE (2021). Role of myeloid-derived suppressor cells in metastasis. Cancer Metastasis Rev.

[109] Gabrilovich DI, Ostrand-Rosenberg S, Bronte V (2012). Coordinated regulation of myeloid cells by tumours. Nat Rev Immunol.

[110] Abe F, Dafferner AJ, Donkor M, Westphal SN (2010). Myeloid-derived suppressor cells in mammary tumor progression in FVB Neu transgenic mice. Cancer Immunol Immunother.

[111] Risom T, Glass DR, Averbukh I, Liu CC (2022). Transition to invasive breast cancer is associated with progressive changes in the structure and composition of tumor stroma. Cell.

[112] Linde N, Casanova-Acebes M, Sosa MS, Mortha A (2018). Macrophages orchestrate breast cancer early dissemination and metastasis. Nat Commun.

[113] Campbell MJ, Baehner F, O'Meara T, Ojukwu E (2017). Characterizing the immune microenvironment in high-risk ductal carcinoma in situ of the breast. Breast Cancer Res Treat.

[114] Toss MS, Abidi A, Lesche D, Joseph C (2020). The prognostic significance of immune microenvironment in breast ductal carcinoma in situ. Br J Cancer.

[115] Semeraro M, Adam J, Stoll G, Louvet E, et al. The ratio of CD8(+)/FOXP3 T lymphocytes infiltrating breast tissues predicts the relapse of ductal carcinoma in situ. Oncoimmunology. 2016;5(10):e1218106-e. 10.1080/2162402X.2016.1218106. 10.1080/2162402X.2016.1218106 PMC5087306 27853639

[116] Miligy I, Mohan P, Gaber A, Aleskandarany MA (2017). Prognostic significance of tumour infiltrating B lymphocytes in breast ductal carcinoma in situ. Histopathology.

[117] Chen X-Y, Thike AA, Md Nasir ND, Koh VCY (2020). Higher density of stromal M2 macrophages in breast ductal carcinoma in situ predicts recurrence. Virchows Arch.

[118] Ubago JM, Blanco LZ, Shen T, Siziopikou KP (2019). The PD-1/PD-L1 axis in HER2+ ductal carcinoma in situ (DCIS) of the breast. Am J Clin Pathol.

[119] Toss MS, Miligy I, Al-Kawaz A, Alsleem M (2018). Prognostic significance of tumor-infiltrating lymphocytes in ductal carcinoma in situ of the breast. Mod Pathol.

[120] Pruneri G, Lazzeroni M, Bagnardi V, Tiburzio GB (2017). The prevalence and clinical relevance of tumor-infiltrating lymphocytes (TILs) in ductal carcinoma in situ of the breast. Ann Oncol.

[121] Bates GJ, Fox SB, Han C, Leek RD (2006). Quantification of regulatory T cells enables the identification of high-risk breast cancer patients and those at risk of late relapse. J Clin Oncol.

[122] Hendry S, Salgado R, Gevaert T, Russell PA (2017). Assessing tumor-infiltrating lymphocytes in solid tumors: A practical review for pathologists and proposal for a standardized method from the International Immunooncology Biomarkers Working Group: Part 1: Assessing the Host Immune Response, TILs in invasive breast carcinoma and ductal carcinoma in situ, metastatic tumor deposits and areas for further research. Adv Anat Pathol.

[123] Dewar R, Fadare O, Gilmore H, Gown AM (2011). Best practices in diagnostic immunohistochemistry: myoepithelial markers in breast pathology. Arch Pathol Lab Med.

[124] Lerwill MF (2004). Current practical applications of diagnostic immunohistochemistry in breast pathology. Am J Surg Pathol.

[125] Liu H (2014). Application of immunohistochemistry in breast pathology: a review and update. Arch Pathol Lab Med.

[126] Jiang Y, Prabakaran I, Wan F, Mitra N (2014). Vav2 protein overexpression marks and may predict the aggressive subtype of ductal carcinoma in situ. Biomark Res.

[127] Hendry S, Pang JB, Byrne DJ, Lakhani SR (2017). Relationship of the breast ductal carcinoma in situ immune microenvironment with clinicopathological and genetic features. Clin Cancer Res.

[128] Hoskoppal D, Reisenbichler ES (2018). Can tumor-associated macrophages in ductal carcinoma in situ (DCIS) on biopsy predict invasive carcinoma on excision?.. Hum Pathol.

